# Modular segregation drives causality of the dynamic oscillatory network responses during threat processing

**DOI:** 10.1093/braincomms/fcad035

**Published:** 2023-02-17

**Authors:** Gabriel Gonzalez-Escamilla, Venkata C Chirumamilla, Nabin Koirala, Abdul R Anwar, Oliver Tüscher, Johannes Vogt, Phillip Horstmann, Benjamin Meyer, George A Bonanno, Sergiu Groppa, Muthuraman Muthuraman

**Affiliations:** Section of Movement Disorders and Neurostimulation, Biomedical Statistics and Multimodal Signal Processing unit, Department of Neurology, Focus Program Translational Neuroscience (FTN), University Medical Center of the Johannes Gutenberg University Mainz, Mainz 55131, Germany; Section of Movement Disorders and Neurostimulation, Biomedical Statistics and Multimodal Signal Processing unit, Department of Neurology, Focus Program Translational Neuroscience (FTN), University Medical Center of the Johannes Gutenberg University Mainz, Mainz 55131, Germany; Section of Movement Disorders and Neurostimulation, Biomedical Statistics and Multimodal Signal Processing unit, Department of Neurology, Focus Program Translational Neuroscience (FTN), University Medical Center of the Johannes Gutenberg University Mainz, Mainz 55131, Germany; Haskins Laboratories, Yale University, New Haven 06511, USA; Biomedical Engineering Centre, University of Engineering & Technology, Lahore (KSK Campus), Lahore, Punjab 39161, Pakistan; Department of Psychiatry and Psychotherapy, University Medical Center of the Johannes Gutenberg University Mainz, Mainz 55131, Germany; Department of Molecular and Translational Neuroscience, Institute of Anatomy II, Cluster of Excellence-Cellular Stress Response in Aging-Associated Diseases (CECAD), Center of Molecular Medicine Cologne (CMMC), University of Cologne, Cologne, Germany; Department of Psychiatry and Psychotherapy, University Medical Center of the Johannes Gutenberg University Mainz, Mainz 55131, Germany; Neuroimaging Center, University Medical Center of the Johannes Gutenberg University Mainz, Mainz 55131, Germany; Department of Clinical Psychology, Teachers College, Columbia University, New York 10027, USA; Section of Movement Disorders and Neurostimulation, Biomedical Statistics and Multimodal Signal Processing unit, Department of Neurology, Focus Program Translational Neuroscience (FTN), University Medical Center of the Johannes Gutenberg University Mainz, Mainz 55131, Germany; Section of Movement Disorders and Neurostimulation, Biomedical Statistics and Multimodal Signal Processing unit, Department of Neurology, Focus Program Translational Neuroscience (FTN), University Medical Center of the Johannes Gutenberg University Mainz, Mainz 55131, Germany

**Keywords:** community structure, threat processing, brain networks, causality, electrophysiology

## Abstract

Physiological responses to threat and stress stimuli entrain synchronized neural oscillations among cerebral networks. Network architecture and adaptation may play a critical role in achieving optimal physiological responses, while alteration can lead to mental dysfunction.

We reconstructed cortical and sub-cortical source time series from high-density electroencephalography, which were then fed into community architecture analysis. Dynamic alterations were evaluated in terms of flexibility, clustering coefficient and global and local efficiency, as parameters of community allegiance. Transcranial magnetic stimulation was applied over the dorsomedial prefrontal cortex during the time window relevant for physiological threat processing and effective connectivity was computed to test the causality of network dynamics.

A theta band-driven community re-organization was evident in key anatomical regions conforming the central executive, salience network and default mode networks during instructed threat processing. Increased network flexibility entrained the physiological responses to threat processing. The effective connectivity analysis showed that information flow differed between theta and alpha bands and were modulated by transcranial magnetic stimulation in salience and default mode networks during threat processing.

Theta oscillations drive dynamic community network re-organization during threat processing. Nodal community switches may modulate the directionality of information flow and determine physiological responses relevant to mental health.

## Teaser

Gonzalez-Escamilla *et al*. report that in the theta band, connectivity dynamics in the central executive, salience and default mode networks change as individuals are exposed to threatening stimuli. They further evidence that networks’ information flow can be modulated with transcranial magnetic stimulation over the dorsomedial prefrontal cortex.

## Graphical Abstract

**Graphical abstract:**
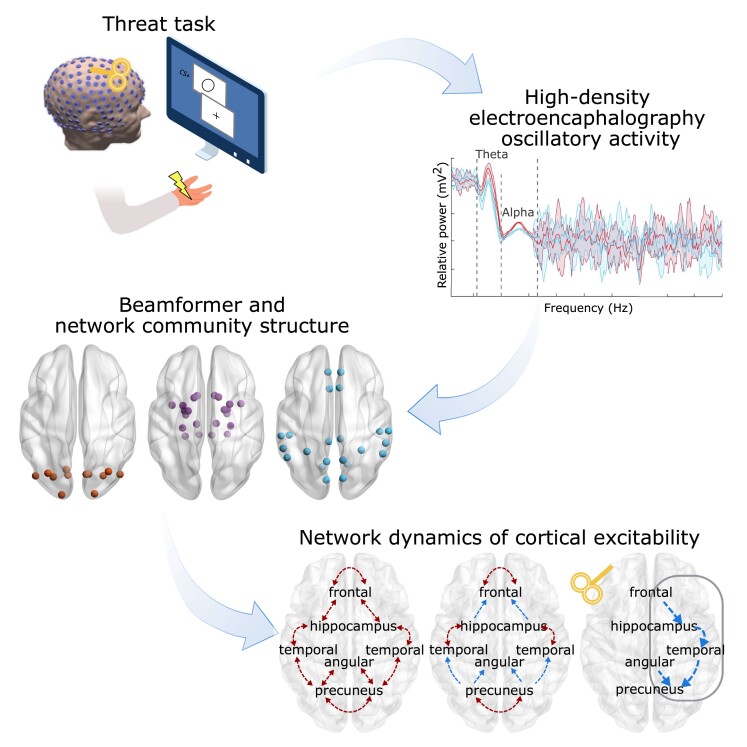

## Introduction

Physiological responses to threat and stress necessitate highly adaptive and orchestrated balance between the functional integration and segregation of distinct networks. Moreover, the appraisal of aversive events involves changes in cognitive states, particularly attention, together with behavioural and physiological responses.^1^ The rapid, temporary shifts on brain excitability upon stressors involve brain core components, in particular the dorsomedial prefrontal cortex (dmPFC), hippocampus and amygdala (AM). These regions are thought to influence primarily adaptive characteristic responses to threat, facilitating coping behaviour.^2^ Furthermore, regions involved in threat processing belong to established networks; for instance, the cingulo-opercular salience network (SN) mediates the detection and integration of behaviourally relevant cognitive, homeostatic or emotional stimuli;^3^ the frontoparietal central executive network (CEN) facilitates self-control as well as reinterpretation of threatening events and emotional information processing;^4^ and the medial prefrontal-parietal default mode network (DMN) enables automated, fast and accurate responses.^5^ Brain oscillations, key elements in the coordination of large-scale brain networks, drive physiological responses to affective stimuli and determine excitability states.^6^ Thus, addressing oscillatory activity within implicated brain networks, in terms of their in-phase synchronization, their states and relation to excitability regulation, could unmask physiological processing dynamics. This would facilitate insights about the individual heterogeneity in adaptation to adverse situations, while abnormalities in network associations could be causally linked to mental disorders.

Although fMRI has been a key tool in characterizing network dynamics during affective processing,^4,7,8^ brain oscillations driving physiological responses occur in the millisecond range. Moreover, distinct neural processes possess a frequency specificity of their evoked responses that cannot be fully captured by fMRI.^9^ Therefore, electroencephalography (EEG) offers the ideal temporal scale to address oscillatory activity related to threat processing within particular networks and, thus, may provide insight into the characterization of the spatiotemporal dynamics of brain networks.^10,11^

Previous evidence suggests that in rodents, oscillations at the theta range (4–8 Hz) support AM–prefrontal coordination and drive physiological threat processing.^12-15^ In human and non-human primates, the emergence of theta oscillations supports the synchronization of AM–prefrontal circuits that serve as mechanism for long-range communication and information transfer during threat processing.^15,16^ In humans, prominent theta power during threat processing in prefrontal, frontal and midline channels has been shown, whereas decrease in alpha activity in parietal and occipital channels occurred.^17-19^ Additionally, oscillations in the alpha range (8–12 Hz) are well-suited for evaluating the sustained anticipatory attention to threat,^20,21^ anticipation^22^ and facilitation of stimulus processing.^23^ Decreases in the attention-related alpha activity have shown to be paralleled by increased cortical excitability,^24,25^ which renders theta and alpha oscillations a potential target for experimental investigations of sustained attentional engagement to threat processing involving neuromodulatory interference such as transcranial magnetic stimulation (TMS).^11^

In the current study, physiological responses to threat processing were evoked using a validated instructed fear paradigm,^11,26-30^ in which a conditioned stimulus (CS+) is paired with an aversive unconditioned stimulus (US). Recent studies indicate that threat responsiveness can be indexed by the presence of the P300, a positive deflection in activity appearing ∼300 ms after the presentation of an attended stimulus allocation and also associated stimulus processing, and the related longer-lasting late positive potential (LPP) components, where the physiological responses to threat processing are depicted as prolonged increased cortical excitability at time intervals around 1000 ms after the stimuli presentation.^11,31-33^ Accordingly, we selected two time points for neuromodulation with TMS: first, before initiation of threat processing and, second, at the physiologically relevant time window. In order to evaluate the causal network dynamics at the EEG temporal resolution, we use a non-linear state–space modelling approach, which uses a dual extended Kalman filtering in a method known as temporal partial directed coherence (TPDC).^34-37^ We hypothesized that looking into simultaneous EEG–TMS data, while modulating threat processing through dmPFC stimulation at distinct time intervals, could uncover local and global network changes at specific neural circuits, specifically CEN, SN and DMN. Network community re-organization at specific oscillations could further modulate the information flow, which we additionally evaluate by observing the directionality of information flow and evaluate if it is time-locked.

The current conceptual framework finds fundament on recent advances in network science.^38^ Particularly, we address the whole-brain network dynamics of physiological threat processing by looking for network community characteristics, which describe functionally specialized sub-networks.^39,40^ Sub-networks are defined as communities of highly interconnected nodes that have very few connections to nodes in other groups. To capture the dynamics of information processing in these network communities, we combine advanced computational algorithms, including regional assignment switching between communities, combined with measures of clustering behaviour (to capture the capacity to form interconnected communities) and flexibility (mirroring the extent to which network regions change their community allegiance over time).^41,42^ The latter can effectively track and quantify the networks’ ability to reconfigure according to task demands.^43^

## Materials and methods

### Study participants

The study included 45 healthy participants (22 females, 23 males, mean age 28 ± 5.48 years). The study protocol was approved by the local ethics committee (Medical Faculty, Johannes Gutenberg University Mainz), and informed written consent was taken from all participants before beginning the experiments. The data was acquired in two different sessions. In the first session, whole-brain magnetic resonance imaging (MRI) data was acquired with various relevant sequences (acquisition parameters and complete experimental procedures are detailed in the Supplementary material). During the second session, participants performed either an instructed threat paradigm (from now on as ‘Experiment 1’; *N* = 19, 11 females, 8 males, mean age 27.4 ± 4.32 years) or an instructed threat paradigm with concurrent TMS (from now on as ‘Experiment 2’; *N* = 26, 11 females, 15 males, mean age 28.6 ± 6.64 years).

‘Ethics approval and consent to participate’: The local ethics committee of the medical faculty of the Johannes Gutenberg University Mainz (Mainz, Germany) approved the study protocol, which is according to the Declaration of Helsinki; all participants provided written informed consent.

### Experiment 1 (instructed threat paradigm)

This experiment was conducted with all participants sitting on a chair and following instructions in the screen. Pain threshold for each participant was obtained by sending out a painful electric stimulus in the dorsal part of left hand using a surface electrode connected to a DS7A electrical stimulator (Digitimer Inc.). Individual pain ratings on a scale from 0 (no pain) to 10 (very unpleasant) were recorded. An intensity representing a pain level of 7 was used during the experiments.

**Figure 1:**
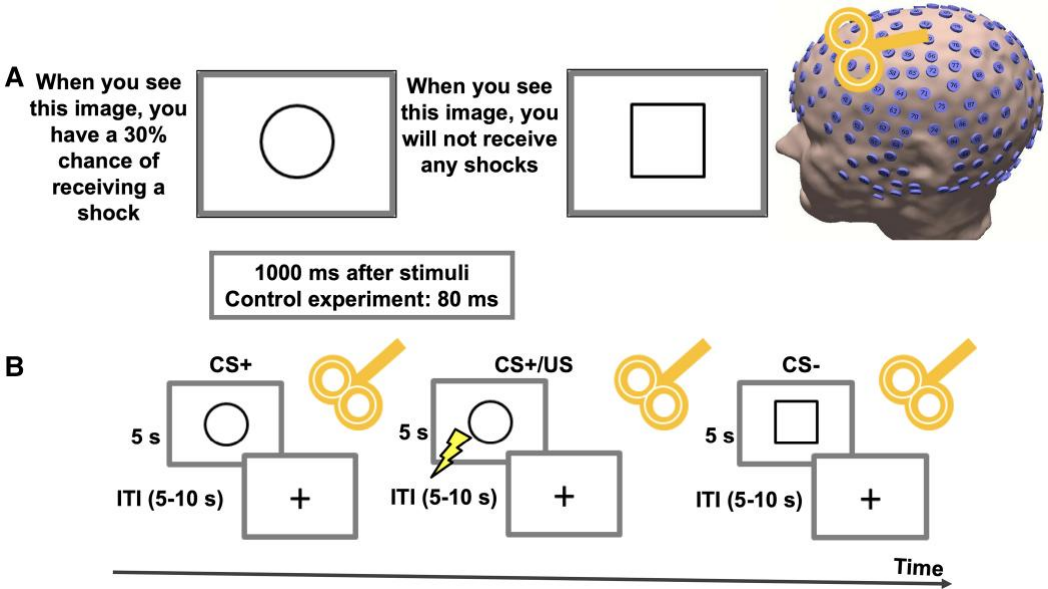
**The schematic representation of the instructed threat paradigm used in this study.** (**A**) The two visual stimuli (symbols) which were used in the instructed threat paradigm and their corresponding explanation: when the circle was presented in screen (CS+), there was a 30% probability to be paired with a threat stimulus (electric shock; US), whereas the square had no stimulus pairing (CS+). (**B**) Example of the temporal scale of the stimuli presentation: each symbol was presented on the computer screen for 5 s followed by a fixation cross with an inter-trial interval (ITI) between 5 and 6 s (Experiment 1). A neuronavigated single-pulse TMS was applied to the right dorsomedial prefrontal cortex (dmPFC) after either 80  or 1000 ms (Experiment 2).

The instructed threat task was administered using the Cogent toolbox (http://www.vislab.ucl.ac.uk/cogent_2000.php) in MATLAB R2006b (MathWorks Inc.). The task consisted of two visual stimuli (circle with and without threat stimuli and square without threat stimuli; Fig. 1A). A fixation cross was shown during the inter-trial interval (ITI) (Fig. 1B). Participants were instructed the screen appearance of a circle (CS+) is associated with a probability of 33% (randomized between 1 and 5 s) of receiving the electric shock (US) of level 7, while screen appearance of a square (CS−) is not associated with any threat. Visual stimuli were presented pseudo-randomly on screen for 5 s, and the ITI was jittered between 4 and 6 s. The paradigm consisted of 60 stimuli (36 CS+, 24 CS−). The experiment was divided in three sessions, where each session lasted around 5 min with 3-min breaks in between sessions. After each session, the level of experienced threat was rated in a scale from 1 to 10 by each participant with a questionnaire. High-density EEG with 256 channels (Net Station 5.0, EGI, USA) was recorded at a sampling frequency of 250 Hz throughout the experiment.

### Experiment 2 (instructed threat paradigm with concomitant dmPFC–TMS

To evaluate the involvement of the dmPFC in threat processing, in this experiment, another cohort of healthy participants undertook the same paradigm as in Experiment 1 together with the application of single TMS pulses over the right hemispheric dmPFC, after 1 s from stimulus onset. The location of the dmPFC for each individual was determined by registering the Montreal Neurological Institute coordinate for the dmPFC ([10 12 58])^44^ to the individual MRI of each participant using SPM8 (http://www.fil.ion.ucl.ac.uk/spm). At the stimulation site, the TMS coil was placed tangentially to the scalp surface and oriented in a medial to lateral position at a 45° angle away from the midline with the handle pointing backwards. The location, position and orientation of the coil were kept unchanged throughout the experiment using a neuro-navigation system (Localite TMS navigator, Germany). TMS pulses were applied in biphasic pulse configuration using a figure-8 coil connected to Magstim Rapid^2^ (Magstim, UK). The intensity of TMS pulses was set to 110% of resting motor threshold. The resting motor threshold was calculated as the minimum stimulus intensity required eliciting motor evoked potentials of amplitude 50 μV in 5 out of 10 consecutive trials at rest.^45^ The paradigm consisted of 90 stimuli (54 CS+, 36 CS−), and subjective threat ratings were acquired. Moreover, the experiment was repeated applying TMS at 80 ms as a control experiment for TMS modulation on the network dynamics. Before preprocessing, condition-specific (CS− and CS+) trials were extracted, and the CS+ trials in which electric shocks were delivered were removed from the data.

### EEG data preprocessing

EEG data preprocessing was performed using MATLAB R2015a and FieldTrip toolbox^46^ in a condition-blind manner. Initially, EEG data was re-referenced to the common grand average reference of all EEG channels and epoched from −2.0 to 4.0 s (0—being the visual stimuli). These epoch trials were used only for the purpose of filtering; for all subsequent analyses, the time interval for the epochs used was −0.25 to 1.5 s. The preprocessing pipeline was adapted from the FieldTrip toolbox as explained in detail in.^17^ For Experiment 1, the EEG data was directly subjected to independent component analyses (FastICA)^47^ to remove the components representing the muscle artefacts, eye blinks, eye movements and line noise. For Experiment 2, firstly a period of −5 to 20 ms of TMS–EEG data (0 as TMS pulse) was removed for avoiding the ringing artefact. The pre-ringing and post-ringing epochs were subject to FastICA to remove the components representing the exponential decay artefact, residual muscle artefacts, eye blinks, eye movements, line noise and other muscle artefacts unrelated to TMS. For Experiment 1, 30 of 256 components (30 ± 4.6, mean ± SD) were rejected where 11 ± 2.68 were related to the eye artefacts, 5 ± 2.34 were related to line noise and 12 ± 1.24 were related to muscle artefacts. Similarly, for Experiment 2, 36 of 256 components (36 ± 2.3) were rejected where 2 ± 0.74 were related to the exponential decay, 4 ± 1.98 were related to line noise, 13 ± 1.16 were related to muscle artefacts and 13 ± 1.04 were related to the eye artefacts. The residual muscle artefacts were visually inspected, removed and interpolated with the cubic interpolation method. A fourth-order Butterworth low-pass filter with a cut-off frequency of 125 Hz was applied to avoid aliasing, which was followed by a band pass filtered between 3 and 45 Hz. Reliability check for EEG signals was performed using the inter-trial phase coherence (ITPC; Supplementary Fig. 1). Individual heart rate was extracted from the EEG signals using the method as detailed in our previous study.^11^ Reconstruction of the brain source activity was based on the finite element modelling from individual MRI and beamformer. The detailed description is found in the Supplementary material. Finally, the difference between CS− and CS+ conditions was computed and used in all subsequent analyses.

### Evaluation of network dynamic re-organization

Based on the reconstructed source activity, individual weighted connectivity matrices were built for theta and alpha bands separately, according to 90 regions defined in the Harvard–Oxford atlas.^48,49^ Connectivity matrix edges represent the theta (or alpha) power cross-correlation between each region of interest (ROI) (j) to all other ROIs(i). Dynamic network topology was then characterized using metrics (see below) from the brain connectivity toolbox^50,51^ and the dynamic graph metrics toolbox.^52^

For each individual connectivity matrix, network communities were firstly identified at baseline (−250 to 0 ms) using the modularity maximization based on Louvain algorithm.^53^ To test the robustness of the detected community associations, we performed 5000 iterations. The final alliance of each ROI to a particular community was based on the maximum number of times-by-iteration the region was assigned to a community.^54,55^ During this process, the resolution parameter (*γ*) was varied (1 to 2.5, in steps of 0.05) to identify a stable and topologically relevant distribution of ROIs in each module. Multilayer modularity maximization depends upon two free parameters, namely, the structural resolution parameter, *γ*, which determines the size of communities: smaller or larger values of *γ* result in correspondingly larger or smaller communities. In this study, we were interested in observing large-scale network alterations during threat processing, hence the resolution parameter, *γ* = 1.65, was selected with larger anatomical modules [(i) frontal; (ii) sensorimotor; (iii) temporal; (iv) occipital; (v) basal ganglia and sub-cortical region; (vi) parietal regions] encompassing the well-established larger functional network. The other parameter, inter-subject coupling parameter, *ω*, which determines the consistency of communities across layers (in our case subjects) with smaller or larger values of *ω* emphasizes community organization that is either unique to individual subjects or shared by the entire cohort, respectively.^56^ Here, the coupling parameter (*ω*) was selected to yield stable six modules across subjects for the resolution parameter selected. For mathematical details about the coupling strength and its computation, please refer to Betzel *et al.*^56^ and Bassett *et al.*^57^ The community assignment was then repeated for six time windows (T1–T6; each 250-ms length), covering the interval from 0 to 1500 ms after the visual stimulus.

### Measures of community efficiency

We assessed four network metrics—flexibility, clustering coefficient and global and local efficiency—characterizing the efficiency of information transfer in all time windows. Node flexibility coefficient reflecting the dynamic community composition^51^ was computed as the number of times the node changes module allegiance through an entire time window of 250 ms (divided into five static windows of 50 ms), normalized by the total possible number of changes (using the Network Community Toolbox (http://commdetect.weebly.com/).^57^ The nodal community flexibility (*f*) could be formulated as:

$$f_{i}=1-\;\frac{1}{T-1}\overset{T-1}{\underset{q=1}{\sum }}\;\delta (Q_{i,q},Q_{i,q+1})$$

Here, *Q* is the output of the multilayer modularity maximization algorithm which detected the community affiliations of each node (ROI) at each window; i.e. the output is *Q = N × T* matrix where each element (*n*, *t*) is the community that node *n* belongs to, at time window *t*. Nodal flexibility is obtained as a number between 0 and 1 where 0 implies most rigidity and 1 implies the most flexibility in terms of community changes through time.^58^ The mean flexibility (*F*) for a subject is then calculated as the mean flexibility score across all nodes as:

$$F=\frac{1}{N}\overset{N}{\underset{i=1}{\sum }}\;f_{i}$$

Further mathematical details could be found in supplementary materials from.^57^ Clustering coefficient (*C*) is a parameter of local organization,^59^ reflecting the number of connections between direct neighbouring nodes with sparsely interconnected regions represented as the abundance of connected triangles in a network, computed for a node *i* as:

$$C_{i}=\frac{\left(Number\;of\;\;connected\;triangles\;including\;node\;i\right)}{k_{i}(k_{i}-1)/2}$$

where $k_{i}=\;\overset{N_{ROI}}{\underset{\;j=1}{\sum }}\;a_{ij}$ and *a*_*ij*_ is the degree of node *i*. Further mathematical details could be found in Rubinov and Sporns^50^ and Masuda, Sakaki, Ezaki and Watanabe.^60^ Global efficiency (*GE*), a measure for network integration reflecting the efficiency of information transfer among all pairs of nodes, is defined as the inverse of the length of the shortest path in the node and computed as:

$$GE=\frac{1}{n}\underset{i\;\in N}{\sum }\;\underset{\;j\in N,j\neq i}{\sum }\;d_{ij}^{-1}$$

where *d* is the topological distance between node *i* and *j*, *N* is the set of all nodes in the network and *n* is the number of nodes.^50^ Please refer to Wang, Ghumare, Vandenberghe and Dupont^61^ and Latora and Marchiori^62^ for detail discussion on mathematical formulation. The local efficiency is the average efficiency of sub-network containing the node and all its immediate neighbours. This primarily reflects how information is exchanged between the neighbours and could measure the network’s fault tolerance.^61,62^ Even though, it provides an important information regarding the network information transfer efficiency, the value obtained for clustering coefficient in an undirected network is reasonably approximate to the local efficiency.^63^ Hence, to avoid redundancy, the local efficiency results are only presented in supplementary materials for interested readers.

For the 3 network parameters, 20 density intervals (range 0.1–0.6) were estimated, over which the mean and standard deviation were computed. The computation of network measures over a range of densities gives the possibility of comparing the network measures where individual networks are not fragmented.^64^

### Investigating causal relationships between network nodes

Evaluation of causal relationships between network nodes (effective connectivity) was effectuated using the TPDC (see complete description in the Supplementary material). This type of non-linear time–frequency causality has been previously applied by our group in both EEG^10,35^ and functional MRI^34,36^ studies.

### Summary of EEG data analyses

In summary, for the EEG data, after preprocessing, source reconstruction was performed using beamformer, followed by power cross-correlation (as measure of connectivity) at the time windows of interest. The resulting connectivity matrices where then used to evaluate dynamic network topology (staring from community detection on baseline and then the variation in community assignment in the following time windows). Network topology for each detected network was evaluated at 20 densities. Finally, we investigated causal relationships between network nodes using the TPDC method.

### Statistical analysis

Data normality was tested using a Shapiro–Wilk test, and sphericity was checked with Mauchly’s sphericity test. The behavioural (threat) ratings and the heart rate estimates were compared between the two stimuli (CS+ and CS−) using paired *t*-tests (at *P* < 0.01).

For each network community and study experiment, the network measures were independently tested using a two-way factorial ANOVA with within-subject factors condition and time. TPDC values between regions tested using a two-way factorial ANOVA with within-subject factors condition and time and *post hoc* were compared using paired *t*-tests. TPDC differences were independently tested at each frequency, each community and each consecutive time window pairs (e.g. baseline versus T1; T1 versus T2; T2 versus T3; T3 versus T4; T4 versus T5; T5 versus T6).

Pearson’s correlation coefficient was estimated between behavioural ratings (CS+ and CS− difference) and the heart rate (CS+ and CS− difference). Finally, network parameters and the effective connectivity values at all pairs of time windows (baseline–T6) were separately correlated with the behavioural ratings and heart rate. Bonferroni correction (*P* < 0.05) was applied.

### Data availability

The raw data are available from the corresponding author upon reasonable request and ethics approval. The data generated and essential to the conclusions of this study is included in the manuscript. The produced code for the time-resolved partial directed coherence (TPDC) can be found in https://github.com/GGonEsc/TPDC. For the rest of analyses, we used open-source toolboxes, including FieldTrip (https://www.fieldtriptoolbox.org/), the Brain Connectivity Toolbox (https://sites.google.com/site/bctnet/) and the Network Community Toolbox (http://commdetect.weebly.com/).

## Results

### Threat state physiological proxy assessment

**Figure 2:**
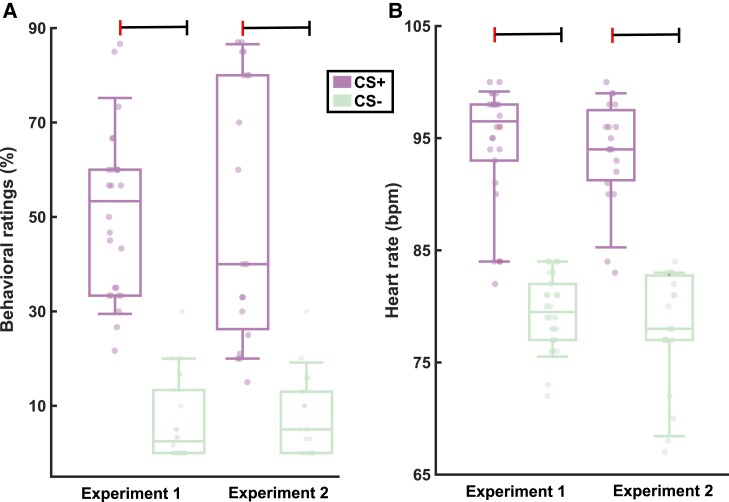
**Behavioural (threat) ratings and the heart rate estimates across experiments.** The boxplots depict the mean and standard deviation for the conditioned stimulus (CS+) and the non-threating stimulus (CS−). For both experiments, paired *t*-tests were employed to compare behavioural ratings (**A**; *P* < 0.001) and heart rate [**B**; beats per minute (bpm); *P* < 0.001]. The dashed line indicates the statistical significance difference between the two stimuli.

In Experiment 1, participants showed higher behavioural threat ratings in the CS+ condition compared to that of those in CS− (*P* < 0.001; Fig. 2A). Heart rates also showed clear increases during CS+ compared to those during CS− (*P* < 0.001; Fig. 2B). Experiment 1 (no TMS) effects were replicated in Experiment 2 (TMS) as increased threatened behaviour (*P* < 0.001; Fig. 2A) and heart rates (*P* < 0.001; Fig. 2B) during CS+. Consistently, correlations between heart rate and threat ratings were attested in both experiments: Experiment 1 (*r* = 0.56; *P* = 0.002) and Experiment 2 (*r* = 0.49; *P* = 0.005).

### Network communities of the source signals

In respect to baseline, the community formation of brain regions varied significantly during processing of the threat. At baseline, six communities with anatomically delimited composition, appeared in both theta and alpha frequency bands. Specifically, Community 1 comprised frontal regions, Community 2, sensorimotor; community 3, temporal; community 4, occipital; community 5, basal ganglia and sub-cortical regions; and community 6, parietal regions. Community alliances were stable over the 5000 iterations, where the regions adhered to the exact same community in 80 ± 6% of the iterations.

**Figure 3:**
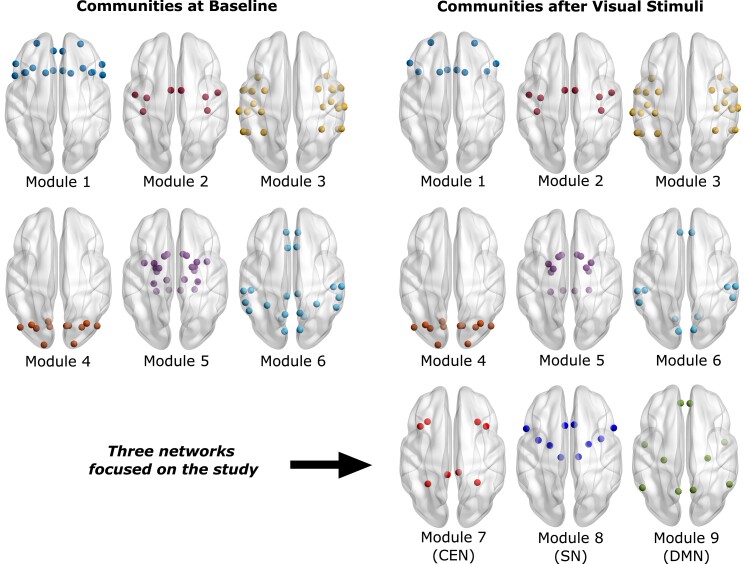
**Network community alliance.** All communities obtained at baseline and after visual stimulus as listed in Table 1. The communities forming after visual stimuli were the central executive network (CEN), salience network (SN) and default mode network (DMN), and thus the analyses were focused on these networks.

After visual stimuli, the same algorithm yielded nine communities for both theta and alpha frequency bands. The additional three communities consisted of nodes from Communities 1, 3, 5 and 6 of the baseline communities, including the brain regions belonging to CEN, SN and DMN. Table 1 shows corresponding brain regions of baseline communities indicating nodes that altered the community alliance, and Fig. 3 visualizes the communities at the baseline and after the visual stimuli.

**Table 1:** Brain regions (as defined in the Harvard–Oxford atlas) are listed based on the community alliance during the baseline window (−250 to 0 ms before visual stimuli presentation)

Community 1	Community 2	Community 3	Community 4	Community 5	Community 6
FP.L-R	PrC.L-R	TP.L-R	LOs.L-R	IC.L-R	SPL.L-R
SFG.L-R	PoC.L-R	STGa.L-R	LOi.L-R	PGa.L-R	SMGa.L-R
MFG.L-R	SMA.L-R	STGp.L-R	ICC.L-R	PGp.L-R	SMGp.L-R
IFGt.L-R	CO.L-R	MTGa.L-R	OFG.L-R	LV.L-R	AG.L-R
IFGo.L-R		MTGp.L-R	SP.L-R	TH.L-R	PCG.L-R
FMC.L-R		MTGto.L-R	OP.L-R	CA.L-R	CGa.L-R
SC.L-R		ITGa.L-R		PU.L-R	CGp.L-R
FO.L-R		ITGp.L-R		PA.L-R	PC.L-R
FOC.L-R		ITGto.L-R		HI.L-R	CC.L-R
		TFCa.L-R		AM.L-R	LG.L-R
		TFCp.L-R		AC.L-R	POC.L-R
		TOF.L-R			
		PP.L-R			
		HG.L-R			
		PT.L-R			

L-R, left and right; FP, frontal pole; SFG, superior frontal gyrus; MFG, middle frontal gyrus; IFGt, inferior frontal gyrus pars triangularis; IFGo, inferior frontal gyrus pars opercularis; FMC, frontal medial cortex; SC, subcallosal cortex; FO, frontal orbital cortex; FOC, frontal operculum cortex; PrC, precentral gyrus; PoC, post central gyrus; SMA, supplementary motor area; CO, central opercular cortex; TP, temporal pole; STGa, superior temporal gyrus anterior division; STGp, superior temporal gyrus posterior division; MTGa, middle temporal gyrus anterior division; MTGp, middle temporal gyrus posterior division; MTGto, middle temporal gyrus temporooccipital; ITGa, inferior temporal gyrus anterior division; ITGp, inferior temporal gyrus posterior division; ITGto, inferior temporal gyrus temporooccipital; TFCa, temporal fusiform cortex anterior division; TFCp, temporal fusiform cortex posterior division; TOF, temporal occipital fusiform cortex; PP, planum polare; HG, Heschl’s gyrus; PT, planum temporale; LOs, lateral occipital superior division; LOi, lateral occipital inferior division; ICC, intracalcarine cortex; OFG, occipital fusiform gyrus; SP, supracalcarine cortex; OP, occipital pole; IC, insular cortex; PGa, parahippocampal gyrus anterior division; PGp, parahippocampal gyrus posterior division; LV, lateral ventricle; TH, thalamus; CA, caudate; PU, putamen; PA, pallidum; HI, hippocampal region; AM, amygdala; AC, accumbens; SPL, superior parietal lobule; SMGa, supramarginal gyrus anterior division; SMGp, supramarginal gyrus posterior division; AG, angular gyrus; PCG, paracingulate gyrus; CGa, cingulate gyrus anterior division; CGp, cingulate gyrus posterior division; PC, precuneus; CC, cuneal cortex; LG, lingual gyrus; POC, parietal operculum cortex.

### Topological characteristics of dynamic community alliance in the theta band

CEN, SN and DMN showed different dynamics in their flexibility, clustering and local efficiency, which were also specific for each frequency band (theta and alpha). Here, we focus on the most relevant results of the theta band. Alpha band results and the results obtained from all densities appear in the Supplementary material and figures.

**Figure 4:**
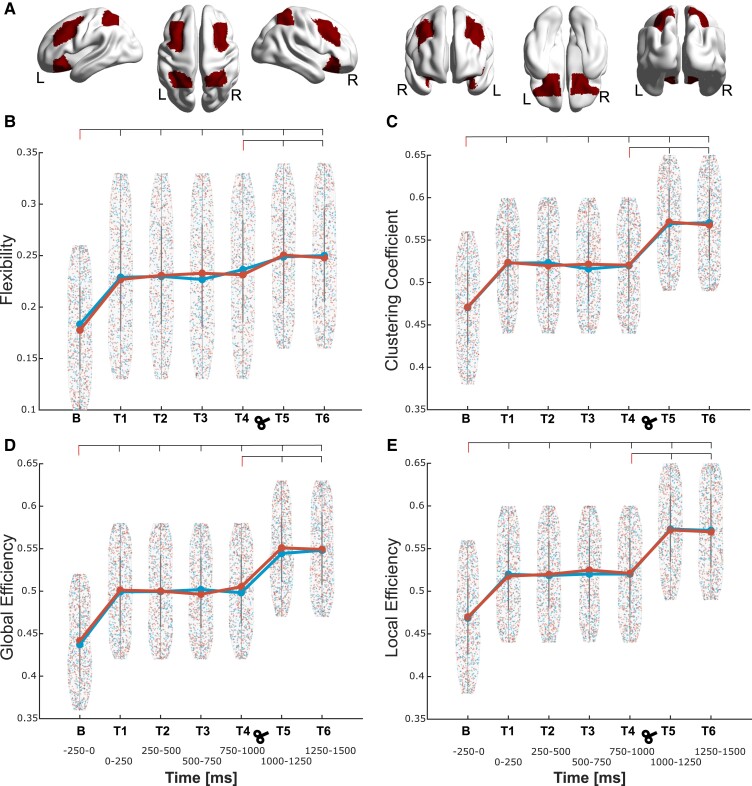
**Topological dynamic characteristics within the central executive network (CEN) in the theta frequency.** (**A**) Depiction of the regions conforming the CEN during threat processing; the corresponding region list is given in Table 1, marked in red colour. (**B**) The network flexibility is shown starting from the baseline (**B**, −250 to 0 ms) window to all the following six time windows (T1–T6, every 250 ms) separately [Experiment 1, factors condition (*F_1,18_* = 15.42, *P* = 0.001) and time (*F_6,108_* = 7.65, *P* < 0.001); Experiment 2, factors condition (*F_1,25_* = 15.53, *P* < 0.001) and time (*F_6,150_* = 8.96, *P* < 0.001)]. **C**, **D** and **E** depict the same as **B** but for clustering coefficient [Experiment 1, factors condition (*F_1,18_* = 16.21, *P* < 0.001) and time (*F_6,108_* = 9.47, *P* < 0.001); Experiment 2, factors condition (*F_1,25_* = 17.24, *P* < 0.001) and time (*F_6,150_* = 7.67, *P* < 0.001)], global efficiency [Experiment 1, factors condition (*F_1,18_* = 10.26, *P* = 0.001) and time (*F_6,108_* = 8.62, *P* < 0.001); Experiment 2, factors condition (*F_1,25_* = 9.28, *P* < 0.001) and time (*F_6,150_* = 7.79, *P* < 0.001)] and local efficiency [Experiment 1, factors condition (*F_1,18_* = 12.31, *P* = 0.001) and time (*F_6,108_* = 9.46, *P* < 0.001); Experiment 2, factors condition (*F_1,25_* = 14.20, *P* < 0.001) and time (*F_6,150_* = 9.35, *P* < 0.001)], respectively. Density plots are presented with data points from ‘Experiment 1’ (without TMS) in blue and ‘Experiment 2’ (with TMS) in red obtained for each density and time window. For each experiment, the mean value across all data points is depicted with a larger circle, while the bars indicate the standard deviation. For each parameter and experiment, the points depict values at each network density for each participant and each of the 20 densities. The reported *F* and *P* values for significant differences between the time intervals were obtained from a two-way factorial ANOVA, and all the intervals were also compared to the baseline for both experiments.

For CEN (Fig. 4A), the theta band showed flexibility increases in both experiments [Fig. 4B; Experiment 1, factors condition (*F_1,18_* = 15.42, *P* = 0.001) and time (*F_6,108_* = 7.65, *P* < 0.001); Experiment 2, factors condition (*F_1,25_* = 15.53, *P* < 0.001) and time (*F_6,150_* = 8.96, *P* < 0.001)]. *Post hoc* analyses confirmed the increased flexibility in all time windows (all *P* < 0.001). This increase indicates the involvement of frontoparietal regions during threat processing and their adaptation to cognitive flexibility. In both experiments, we aimed at evaluating the sustained responses to threat, regarded as increased cortical excitability at time intervals around 1000 ms. Accordingly, T5 (1000–1250 ms after stimuli presentation) showed even higher flexibility (*P* < 0.001) with respect to T4 (750–1000 ms after stimuli presentation). In addition, the increase of network flexibility in the T5 shows a change in the physiological theta after 1 s, which is the indication that the sustained flexibility is necessary during threat processing.

CEN theta band clustering coefficient (Fig. 4C) showed increases in both experiments [Experiment 1, factors condition (*F_1,18_* = 16.21, *P* < 0.001) and time (*F_6,108_* = 9.47, *P* < 0.001); Experiment 2, factors condition (*F_1,25_* = 17.24, *P* < 0.001) and time (*F_6,150_* = 7.67, *P* < 0.001)]. *Post hoc* analyses evidenced significant increases in all time intervals (all *P* < 0.001). In both experiments, T5 was higher than T4 (*P* < 0.001). The increase in cognitive flexibility in these particular time windows T1 and T5 go hand in hand with the increase in clustering coefficient due to high density of relatively short axons and the close proximity of nodes within each module.

The global efficiency (Fig. 4D) of the theta band in CEN was also increased [Experiment 1, factors condition (*F_1,18_* = 10.26, *P* = 0.001) and time (*F_6,108_* = 8.62, *P* < 0.001); Experiment 2, factors condition (*F_1,25_* = 9.28, *P* < 0.001) and time (*F_6,150_* = 7.79, *P* < 0.001)], where the *post hoc* analyses revealed the increases in all time windows (all *P* < 0.001). *Post hoc* analyses evidenced significant increases in all time intervals (all *P* < 0.001). In both experiments, T5 was increased compared to T4 (*P* < 0.001). The efficiency in this network was increased at T1 compared to baseline, indicating the alertness of the brain to aversive stimuli.

The local efficiency (Fig. 4E) of the theta band in CEN was also increased [Experiment 1, factors condition (*F_1,18_* = 12.31, *P* = 0.001) and time (*F_6,108_* = 9.46, *P* < 0.001); Experiment 2, factors condition (*F_1,25_* = 14.20, *P* < 0.001) and time (*F_6,150_* = 9.35, *P* < 0.001)], where the *post hoc* analyses revealed the increases in all time windows (all *P* < 0.001). However, only in Experiment 2 (TMS), local efficiency was increased in T5 compared to T4 (*P* < 0.001). The increase in local information transfer during threat processing, reflected by the increase in clustering within modules, could be either up- or downregulation of the network, based on distinct synchronized oscillations.

**Figure 5:**
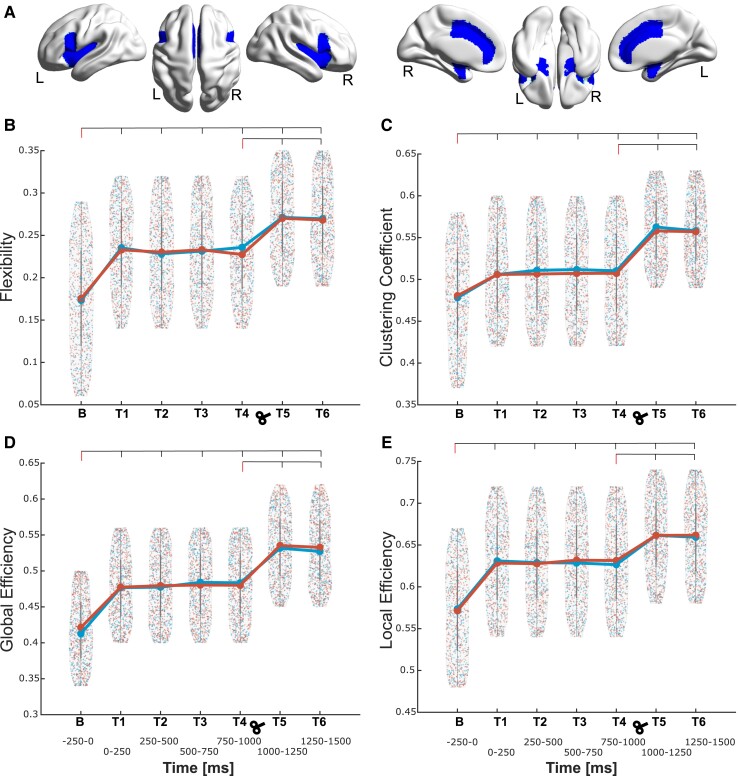
**Topological dynamic characteristics within the salience network (SN) in the theta frequency.** (**A**) Depiction of the regions conforming the SN; the corresponding region list is given in Table 1, marked in blue colour. (**B**) The network flexibility is shown starting from the baseline (**B**, −250 to 0 ms) window to all the following six time windows (T1–T6, every 250 ms) separately [Experiment 1, factors condition (*F_1,18_* = 12.67, *P* = 0.002) and time (*F_6,108_* = 4.24, *P* = 0.001); Experiment 2 (factors condition (*F_1,25_* = 9.24, *P* = 0.006) and time (*F_6,150_* = 5.87, *P* < 0.001)]. Plots at **C**, **D** and **E** depicts the network flexibility starting from baseline to all six time windows for clustering coefficient, Global and local efficiency respectively. Clustering coefficient [Experiment 1, factors condition (*F_1,18_* = 10.54, *P* = 0.005) and time (*F_6,108_* = 3.64, *P* = 0.008); Experiment 2, factors condition (*F_1,25_* = 8.46, *P* = 0.009) and time (*F_6,150_* = 3.72, *P* = 0.008)], global efficiency [Experiment 1, factors condition (*F_1,18_* = 10.54, *P* = 0.005) and time (*F_6,108_* = 3.64, *P* = 0.008); Experiment 2, factors condition (Experiment 1, factors condition (*F_1,18_* = 7.41, *P* = 0.001) and time (*F_6,108_* = 5.65, *P* < 0.001); Experiment 2, factors condition (*F_1,25_* = 5.31, *P* = 0.004) and time (*F_6,150_* = 6.27, *P* < 0.001)] and local efficiency [Experiment 1, factors condition (*F_1,18_* = 11.25, *P* = 0.003) and time (*F_6,108_* = 4.78, *P* < 0.001); Experiment 2, factors condition (*F_1,25_* = 11.45, *P* = 0.002) and time (*F_6,150_* = 5.46, *P* < 0.001)]. Density plots are presented with data points from ‘Experiment 1’ (without TMS) in blue and ‘Experiment 2’ (with TMS) in red obtained for each density and time windows. For each experiment, the mean value across all data points is depicted with a larger circle, while the bars indicate the standard deviation. For each parameter and experiment, the points depict values at each network density for each participant and each of the 20 densities. The reported *F* and *P* values for significant differences between the time intervals were obtained from a two-way factorial ANOVA, and all the intervals were also compared to the baseline for both experiments.

For SN (Fig. 5A), flexibility in the theta band (Fig. 5B) appeared increased in both experiments [Experiment 1, factors condition (*F_1,18_* = 12.67, *P* = 0.002) and time (*F_6,108_* = 4.24, *P* = 0.001); Experiment 2 (factors condition (*F_1,25_* = 9.24, *P* = 0.006) and time (*F_6,150_* = 5.87, *P* < 0.001)]. *Post hoc* analyses attested these effects for T1–T6 compared to baseline (all *P* < 0.001). Concordantly, in both experiments, T5 showed increased flexibility compared to that of T4 (*P* < 0.001). This indicates that the connectivity between sub-cortical structures and cortical regions plays a significant role in cognitive flexibility during the processing of threat.

SN theta band clustering coefficient (Fig. 5C) showed significant increases in both experiments [Experiment 1, factors condition (*F_1,18_* = 10.54, *P* = 0.005) and time (*F_6,108_* = 3.64, *P* = 0.008); Experiment 2, factors condition (*F_1,25_* = 8.46, *P* = 0.009) and time (*F_6,150_* = 3.72, *P* = 0.008)]. *Post hoc* analyses confirmed the increased clustering in all T1–T6 compared to baseline (all *P* < 0.01). Here, only in Experiment 2, T5 showed increased clustering compared to T4 (*P* < 0.001).

SN theta band global efficiency (Fig. 5D) was also increased in both experiments [Experiment 1, factors condition (*F_1,18_* = 7.41, *P* = 0.001) and time (*F_6,108_* = 5.65, *P* < 0.001); Experiment 2, factors condition (*F_1,25_* = 5.31, *P* = 0.004) and time (*F_6,150_* = 6.27, *P* < 0.001)]. *Post hoc* analyses revealed differences at all time windows with respect to baseline (all *P* < 0.01). Global efficiency increases were detected for the interval T5 compared to T4 in both experiments (*P* < 0.01). The results indicate that the global information transfer, largely restrained by the thalamus, in cortical regions increases during threat processing.

SN theta band local efficiency (Fig. 5E) was increased in both experiments [Experiment 1, factors condition (*F_1,18_* = 11.25, *P* = 0.003) and time (*F_6,108_* = 4.78, *P* < 0.001); Experiment 2, factors condition (*F_1,25_* = 11.45, *P* = 0.002) and time (*F_6,150_* = 5.46, *P* < 0.001)]. *Post hoc* analyses revealed differences at all time windows with respect to baseline (all *P* < 0.01). Local efficiency increases were detected for the interval T5 compared to T4 in both experiments (*P* < 0.01). The local information transfer could indicate the increase in connectivity in sub-cortical to cortical loop to maintain the cognitive flexibility across the network.

**Figure 6:**
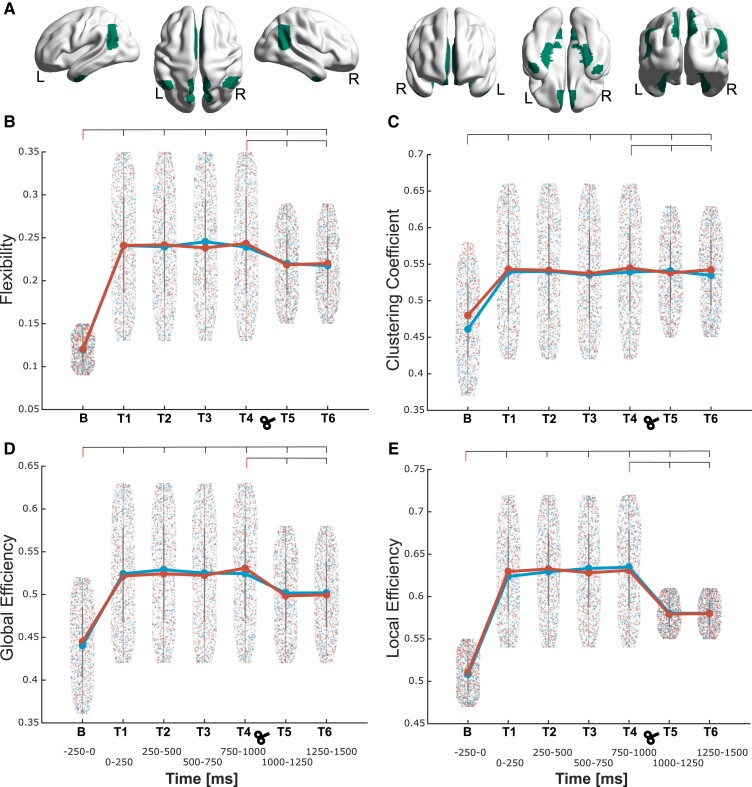
**Topological dynamic characteristics within the default mode network (DMN) in the theta frequency.** In **A**, the representative figure with regions comprised in the DMN; the corresponding list is given in Table 1, marked in green colour. (**B**) The network flexibility is shown starting from the baseline (**B**, −250 to 0 ms) window to all the following six time windows (T1–T6, every 250 ms) separately [Experiment 1, factors condition (*F_1,18_* = 22.67, *P* < 0.001) and time (*F_6,108_* = 12.24, *P* < 0.001); Experiment 2, factors condition (*F_1,25_* = 20.45, *P* < 0.001) and time (*F_6,150_* = 14.87, *P* < 0.001)]. Plots at **C, D** and **E** depicts the network flexibility starting from baseline to all six time windows for clustering coefficient, Global and local efficiency respectively. Clustering coefficient [Experiment 1, factors condition (*F_1,18_* = 38.74, *P* < 0.001) and time (*F_6,108_* = 19.57, *P* < 0.001); Experiment 2, factors condition (*F_1,25_* = 34.21, *P* < 0.001) and time (*F_6,150_* = 17.24, *P* < 0.001)], global efficiency [Experiment 1, factors condition (*F_1,18_* = 10.12, *P* < 0.001) and time (*F_6,108_* = 6.98, *P* < 0.001); Experiment 2, factors condition (*F_1,25_* = 8.46, *P* < 0.001) and time (*F_6,150_* = 11.27, *P* < 0.001)] and local efficiency [Experiment 1, factors condition (*F_1,18_* = 42.28, *P* < 0.001) and time (*F_6,108_* = 23.27, *P* < 0.001); Experiment 2, factors condition (*F_1,25_* = 39.65, *P* < 0.001) and time (*F_6,150_* = 19.38, *P* < 0.001)]. Density plots are presented with data points from ‘Experiment 1’ (without TMS) in blue and ‘Experiment 2’ (with TMS) in red obtained for each density and time windows. For each experiment, the mean value across all data points is depicted with a larger circle, while the bars indicate the standard deviation. For each parameter and experiment, the points depict values at each network density for each participant and each of the 20 densities. The reported *F* and *P* values for significant differences between the time intervals were obtained from a two-way factorial ANOVA, and all the intervals were also compared to the baseline for both experiments.

For the DMN (Fig. 6A), an increased flexibility in the theta band was observed [Fig. 6B; Experiment 1, factors condition (*F_1,18_* = 22.67, *P* < 0.001) and time (*F_6,108_* = 12.24, *P* < 0.001); Experiment 2, factors condition (*F_1,25_* = 20.45, *P* < 0.001) and time (*F_6,150_* = 14.87, *P* < 0.001)]. *Post hoc* analyses showed that higher flexibility occurred in all time windows after stimuli presentation in comparison to baseline (all *P* < 0.001). However, in both experiments, T5 (1000–1250 ms) showed a decrease in flexibility (*P* < 0.001) compared to that of T4 (750–1000 ms).

Also for DMN, clustering coefficient was also increased in the theta band (Fig. 6C) in both experiments [Experiment 1, factors condition (*F_1,18_* = 38.74, *P* < 0.001) and time (*F_6,108_* = 19.57, *P* < 0.001); Experiment 2, factors condition (*F_1,25_* = 34.21, *P* < 0.001) and time (*F_6,150_* = 17.24, *P* < 0.001)]. *Post hoc* analyses revealed significant differences between baseline and all T1–T6 windows (all *P* < 0.001).

DMN theta band global efficiency (Fig. 6D) was increased in both experiments [Experiment 1, factors condition (*F_1,18_* = 10.12, *P* < 0.001) and time (*F_6,108_* = 6.98, *P* < 0.001); Experiment 2, factors condition (*F_1,25_* = 8.46, *P* < 0.001) and time (*F_6,150_* = 11.27, *P* < 0.001)]. Here, a decreased local efficiency was observed for T5 in comparison to T4 in both experiments (*P* < 0.001).

DMN theta band local efficiency (Fig. 6E) was increased in both experiments [Experiment 1, factors condition (*F_1,18_* = 42.28, *P* < 0.001) and time (*F_6,108_* = 23.27, *P* < 0.001); Experiment 2, factors condition (*F_1,25_* = 39.65, *P* < 0.001) and time (*F_6,150_* = 19.38, *P* < 0.001)]. Here, decreased local efficiency was observed for T5 in comparison to that for T4 in both experiments (*P* < 0.001). Interestingly, in this modular network which is involved in a background physiological processing, the flexibility, global efficiency and the local information transfer followed the other two modules by an increase. However, the relatively longer physiological processing found in other modules (CEN and SN) was reduced in this module indicating a more compensatory processes for maintaining the equilibrium.

The corresponding topology metrics of the SN and DMN in the alpha band are found in Supplementary Figs. 2 and 3. For all three networks, the mean value across participants in the theta band is depicted in Supplementary Figs. 4–6. No effects in flexibility, clustering and local efficiency were found for the control experiment (single-pulse TMS at 80 ms) in all the three communities (Supplementary Fig. 7).

### Information flow dynamics during threat processing

The effective connectivity analyses focused on the difference between the two conditions (CS+ and CS−) within the three threat-related communities. Here, only information flows that survived surrogate time reversal (*P* < 0.001) for theta and alpha bands are reported.

**Figure 7:**
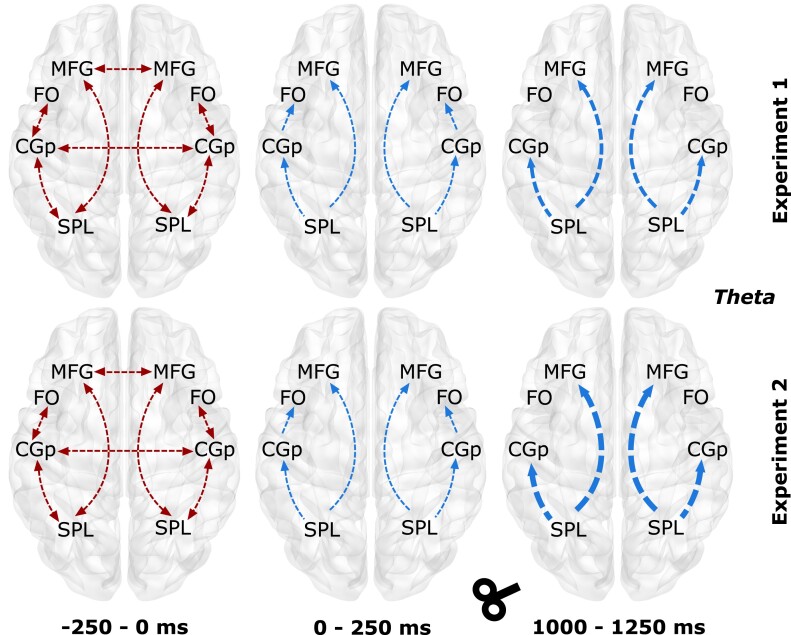
**Temporal information flow dynamics across the regions in the central executive network (CEN) within the theta band.** The results from temporal partial directed coherence (TPDC) are shown over the template brain. The two rows represent Experiments 1 and 2 for the theta frequency band. The red arrows indicate the information flow during the baseline window (**B**, −250 to 0 ms), and the blue lines indicate the difference in the information flow with the previous time window. The arrow thickness indicates the strength of the information flow when compared to the previous time window. Please refer to Table 1 legend for full forms of the abbreviations in the figure. CGp, cingulate gyrus posterior division; FO, frontal orbital cortex; MFG, middle frontal gyrus; SPL, superior parietal lobule.

In CEN (Fig. 7), the theta band effective connectivity (EC) increased in both experiments [Experiment 1, factors condition (*F_1,18_* = 5.38, *P* = 0.0086) and time (*F_6,108_* = 4.29, *P* = 0.0074); Experiment 2, factors condition (*F_1,25_* = 6.21, *P* = 0.0063) and time (*F_6,150_* = 5.74, *P* = 0.0081)]. The information flow of the baseline window was replicated in both experiments, was bi-directional and was restricted to parieto-frontal regions. At T1 (after stimuli appearance), all connectivity changed from bi-directional to uni-directional. At T5 (after TMS), fewer connections showed the increase in EC, but the connectivity was strengthened, specifically SPL to MFG and SPL to CGp.

**Figure 8:**
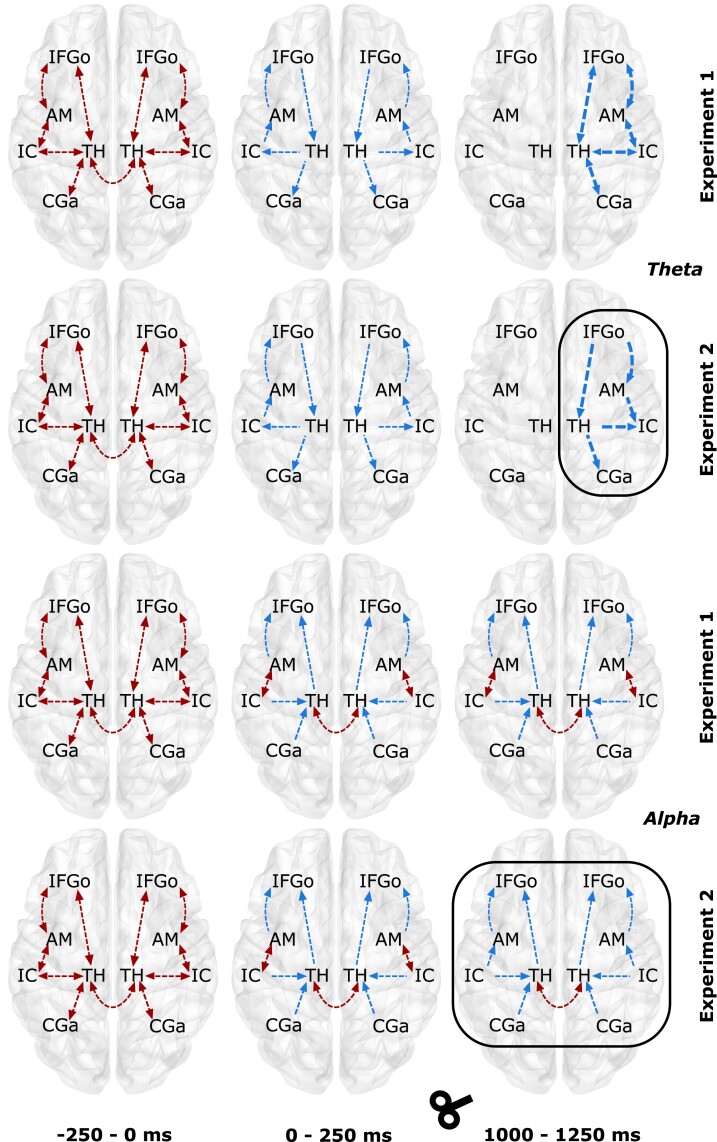
**Temporal information flow dynamics across the regions in the salience network (SN) within theta and alpha bands.** The results from temporal partial directed coherence (TPDC) are shown over the template brain. The first two rows represent Experiments 1 and 2 for the theta frequency band whereas last two rows represent the same for alpha band. The red arrows indicate the information flow during the baseline window (**B**, −250 to 0 ms), and the blue lines indicate the difference in the information flow with the previous time window. The arrow thickness indicates the strength of the information flow when compared to the previous time window. The black box indicates the difference in information flow between Experiment 1 and Experiment 2. Please refer to Table 1 legend for full forms of the abbreviations in the figure. AM, amygdala; CGa, cingulate gyrus anterior division; IC, insular cortex; IFGo, inferior frontal gyrus pars opercularis; TH, thalamus.

For SN (Fig. 8), the theta band, EC significantly increased in both experiments [Experiment 1, factors condition (*F_1,18_* = 10.65, *P* < 0.001) and time (*F_6,108_* = 3.68, *P* = 0.0096); Experiment 2, factors condition (*F_1,25_* = 11.46, *P* < 0.001) and time (*F_6,150_* = 3.24, *P* = 0.023)]. During baseline, only bi-directional connections were observed in both experiments, which were mostly intra-hemispheric. At T1, the connectivity was largely uni-directional with bilateral thalamus connectivity missing. At T5, connectivity was restricted to the right hemispheric regions, and EC was strengthened. Most notably, TMS modulation of the theta band (T5 in Experiment 2) caused the existing bi-directional EC (frontal-AM), frontal-thalamus and AM-insular cortex (IC) to be uni-directional. The alpha band EC at baseline was similar to that observed in theta band. We also found significant changes for both factors in the alpha band [Experiment 1, factors condition (*F_1,18_* = 10.47, *P* < 0.001) and time (*F_6,108_* = 4.12, *P* = 0.0046); Experiment 2, factors condition (*F_1,25_* = 9.52, *P* < 0.001) and time (*F_6,150_* = 2.98, *P* = 0.033)]. At T1, most of the connectivity turned uni-directional, but few remained bi-directional (IC–AM and bilateral thalamus). In Experiment 1, no change in connectivity was observed at T;, however, in Experiment 2, all except bilateral thalamus connectivity turned uni-directional.

**Figure 9:**
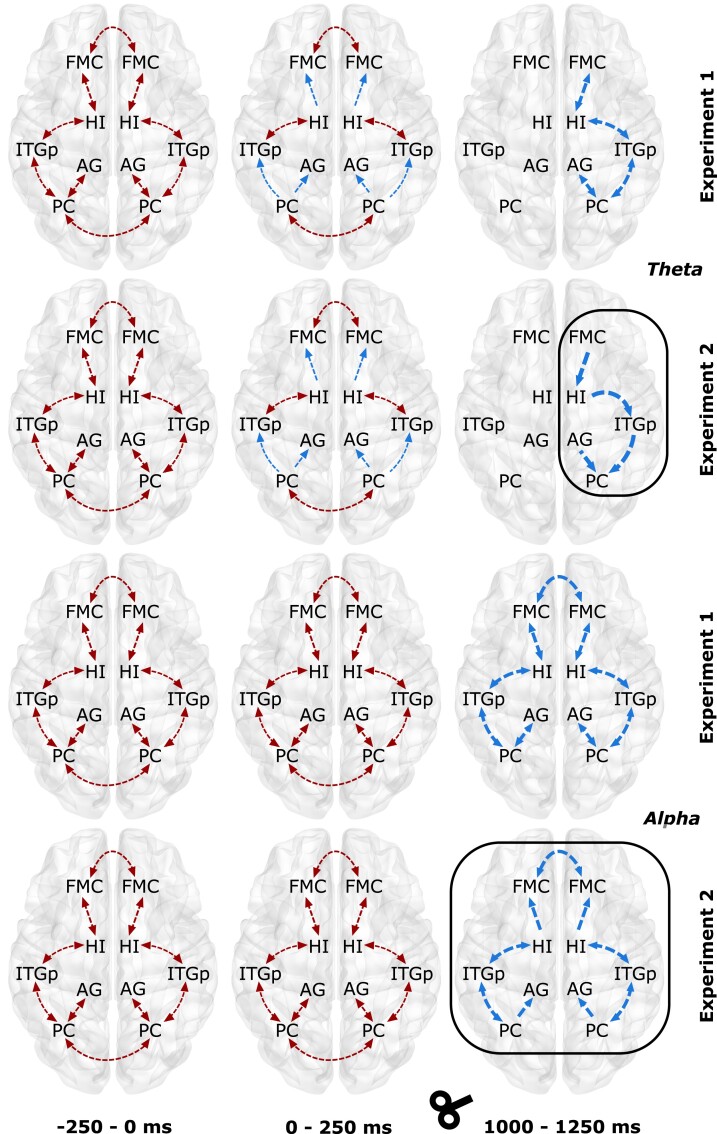
**Temporal information flow dynamics across the regions in the default mode network (DMN) within theta and alpha bands.** The results from temporal partial directed coherence (TPDC) are shown over the template brain. The first two rows represent Experiments 1 and 2 for the theta frequency band whereas the last two rows represent the same for alpha band. The red arrows indicate the information flow during the baseline window (**B**, −250 to 0 ms), and the blue lines indicate the difference in the information flow with the previous time window. The arrow thickness indicates the strength of the information flow when compared to the previous time window. The black box indicates the difference in information flow between Experiment 1 and Experiment 2. Please refer to Table 1 legend for full forms of the abbreviations in the figure. AG, angular gyrus; FMC, frontal medial cortex; HI, hippocampal; ITGp, inferior temporal gyrus posterior division; PC, precuneus.

For DMN (Fig. 9), the theta band EC increased significantly in both experiments [Experiment 1, factors condition (*F_1,18_* = 12.79, *P* < 0.001) and time (*F_6,108_* = 4.98, *P* = 0.0016); Experiment 2, factors condition (*F_1,25_* = 15.38, *P* < 0.001) and time (*F_6,150_* = 5.46, *P* = 0.0023)]. The connectivity at baseline was only bi-directional for both intra and inter-hemispheric connections. At T1, the EC remained unaltered between ITGp–HI, bilateral FMC and PC but changed to uni-directional for those between PC–ITGp, PC–AG and HI–FMC. At T5, in Experiment 1, all connections were restricted to the right hemisphere, and the connectivity was bi-directional; however, in Experiment 2, TMS theta modulation was evident in not only strengthening the connections but also changing the directionality to uni-directional. For alpha band EC, baseline connectivity was same as that in theta band and we also found significant increase in EC for both the factors [Experiment 1, factors condition (*F_1,18_* = 7.45, *P* < 0.001) and time (*F_6,108_* = 3.87, *P* = 0.0039); Experiment 2, factors condition (*F_1,25_* = 6.48, *P* < 0.001) and time (*F_6,150_* = 3.05, *P* = 0.029)]. At T1, the connectivity remained unaltered from baseline for both experiments. However, at T5, in Experiment 1, the connectivity strengthened for all connections, and for Experiment 2, in addition to the strengthening of the connectivity, the directionality for connectivity between HI–FMC and PC–AG changed from bi-directional to uni-directional.

### Correlations between electrophysiological and behavioural indicators of threat processing

In the theta band, we found a correlation between baseline–T1 (frontal ITPC; baseline referenced T1) and the heart rate in both experiments [Experiment 1 (*r* = 0.70; *P* = 0.014); Experiment 2 (*r* = 0.61; *P* = 0.012)]. For the network parameters in the theta band of CS+, a correlation was also found for the baseline–T1 between flexibility and heart rate, for SN and DMN in both experiments [Experiment 1 (*r* = 0.68; *P* = 0.003; *r* = 0.56; *P* = 0.005); Experiment 2 (*r* = 0.58; *P* = 0.005; *r* = 0.61; *P* = 0.004)]. For effective connectivity (Table 2), a correlation between baseline connectivity and the threat ratings (difference CS+/CS−) was significant for SN; specifically, for left IC–left AM (*r* = 0.40; *P* = 0.006) and left IFGo–left TH (*r* = 0.37; *P* = 0.009).

**Table 2:** Significant correlations identified in the three newly formed communities between the theta band effective connectivity and heart rate in Experiments 1 and 2

Community	Connection	R	*P*
Experiment 1			
CEN	SPL (left) to CGp (left)	0.624	0.0002
SN	TH (right) to IC (right)	0.587	0.0003
Experiment 2			
CEN	SPL (left) to CGp (left)	0.684	0.0003
SN	TH (right) to IC (right)	0.387	0.004

SN, salience network; CEN, central executive network; SPL, superior parietal lobule; CGp, cingulate gyrus posterior division; IC, insular cortex; TH, thalamus.

In the alpha band, for both experiments correlations where found only with effective connectivity for the baseline–T1 (Table 3). The correlation results for theta band effective connectivity at T5 with heart rate are listed in (Table 4).

**Table 3:** The significant correlations identified in the three newly formed communities between the alpha band effective connectivity and heart rate in Experiments 1 and 2

Community	Connection	*r*	*P*
Experiment 1			
SN	TH (left) to IFGo (left)	−0.457	0.003
SN	IC (right) to AM (right)	−0.398	0.007
Experiment 2			
SN	IC (left) to AM (left)	−0.464	0.005
SN	TH (left) to IFGo (left)	−0.376	0.008

SN, salience network; SOG, superior occipital gyrus; MOG, middle occipital gyrus; IOG, inferior occipital gyrus; PCG, posterior cingulate gyrus; ANG, angular gyrus; SMA, supplementary motor area; INS, insula; AMYG, amygdala; PUT, putamen; THA, thalamus.

**Table 4:** The significant correlations were found only in Experiment 2 between the three newly formed communities theta frequency band connectivity values and heart rate

Experiment 2			
Community	Connection	*r*	*P*
SN	IFGo (right) to AM (right)	0.672	0.0002
DMN	HI (right) to ITGp (right)	0.647	0.0003
DMN	FMC (right) to HI(right)	0.587	0.0002
SN	AM (right) to IC (right)	0.524	0.002

SN, salience network; DMN, default mode network; MFG, middle frontal gyrus; SMA, supplementary motor area; INS, insula; AMYG, amygdala; HIPP, hippocampus; STG, superior temporal gyrus; ORBmid, middle frontal gyrus, orbital part.

## Discussion

Taken together, our data revealed the key role of theta oscillations in the dynamics of network re-organization during physiological responses to aversive stimuli. We evidenced that physiological threat processing requires the transition between cognitive-free periods to associative learning, which relies on CEN, SN and DMN circuits. Within these network communities, we evidenced a time-dependent threat-induced network behaviour (increased flexibility and clustering in the SN), which can be causally modulated by application of TMS pulse over dMPFC, 1000 ms after CS+ presentation. We based the timing of the TMS pulses to dMPFC on the dynamics of theta driven alterations, which return to baseline at 1000 ms, as shown by inter-trial coherence in the experiment without TMS. To confirm our analyses, a second TMS pulse over dMPFC was applied at a time period not relevant for threat processing. This pulse had no influence with the ongoing network behaviour and community restructuring.

### Dynamic community re-organization is required for threat processing

Concordant with previous results,^65,66^ we show that baseline activity (before CS+ occurrence) has a functional community composition. However, threat presentation causes the community composition to be reconfigured into three well-known networks: CEN, SN and DMN. Moreover, small-world architecture was evident in these three modules. The small-world network organization indicates that the brain could sustain higher communication efficiency across brain regions with lower energy consumption.^59,67^ The relationship between small-world architecture in relation to threat response has only recently emerged suggesting that the interplay between synchrony of oscillations and the network architecture is a key factor to mediate and sustain efficient information transfer for a longer time periods.^68^ However, the state-dependent dynamics of the network, especially its dependency to stimuli relevance, remained poorly understood. Thus, restructuring from resting- to task-related networks appears to be a primordial mechanism that mediates perception of relevant inputs and subsequent higher-order processing. The involvement of CEN, SN and DMN networks and their core components has been independently described for aversive processing,^3,69-71^ whereas recent studies have proposed that interactions between these networks facilitate cognitive control and aversive processing.^70,72,73^ Of notice, the DMN has positive contributions to the performance of externally directed, attention-demanding, goal-oriented, non-self-referential tasks that require cognitive flexibility to contribute to adaptive behavioural responses.^5^ Here, the network flexibility emerges as a state-dependent component of the threat processing, evidenced by its increase in the three networks theta band. Network flexibility has been already shown to increase according to task demands when cognitive flexibility is required,^57,74^ suggesting that dynamic reconfiguration of brain networks boosts efficient threat processing.

In our study, the fact that participants are aware of the contingency between CS+ and US, i.e. they expect the threatening event,^75^ is of relevance since this likely involves the recruitment of additional resources (attentional and control) to those needed during classical Pavlovian threat conditioning.^11,71,76^ Our results confirm this hypothesis by highlighting the presence of parallel mechanisms during threat processing, where the involved networks (DMN, SN and CEN) may simultaneously endure different aspects of high-order cognitive workflow in order to cope with the situation, for instance, attention, working memory, self-control and emotional regulation. In a recent fMRI study, negative emotional processing network showed tendency to form modular structure and small-world properties with increased local processing.^68^ Other studies have further highlighted segregated modules for different functional task (medial, visual, temporal, sub-cortical) and for DMN.^65,66^ The stress-related network encompassing salience and executive control network is reported either to be upregulated or downregulated depending upon the specific scenario.^77^ However, online interactions of these networks during stress have not been yet possible because of the limited temporal resolution of the MR imaging.

### Information flow directionality is key for threat processing and its behavioural correlates

The present results further evidence causal network dynamics within the reconfigured networks during threat processing, which are accessible to neuromodulation with TMS. The temporal changes in information flow to threat processing turned predominantly uni-directional, which correspond to AM–hippocampus low-frequency oscillation dynamics.^78^ More specifically, during threat processing, CEN and SN connectivity turned into a dominantly uni-directional pattern, involving parieto-frontal regions together with the AM and hippocampus. The temporal changes associated to threat processing are predominantly mediated uni-directionally.^79^ Here, we showed that the connectivity in the DMN take a more parietal to frontal uni-directional route to regulate the threat processing, which is consistent with previous results using classical Pavlovian fear conditioning paradigms.^80^ In addition, we perturbed the network by applying TMS over the dmPFC, showing its ability to modulate the flexibility and the dynamical local information transfer in the network. Previous research have suggested the relation between the connectivity of the core areas belonging to the SN exists and drives the increase in physiological responses during threat processing.^81^ Our data adds to these findings, demonstrating more uni-directional and stronger connectivity in the SN during response to threat processing and TMS perturbations and an increased information flow from the frontal to sensorimotor and thalamic regions. Similar heightened response of the executive control for predictable threat stimuli has been previously demonstrated using startle responses.^76^ The heightened local modulation in the fear network found in our study with increased bi-directional connections between the core regions triggered by TMS stimulation is supported by previous findings showing a large-scale network alterations^82-84^ and modulation of local and global cortical regions^85,86^ during TMS stimulation.

We test whether targeted modulation of a specific region through TMS can hamper network dynamics, as previously suggested^82-84^ for modulating re-organization and information flow among distant regions.^85,86^ In this case, we selected a region highly involved on threat processing, i.e. the mPFC.^11,75^ Behavioural responses have been shown to be good correlates of induced threat processing.^75,87^ Accordingly, significant increases for the CS+ in threat ratings and heart rate were observed in our experiments; however, concordant with previous reports,^88,89^ neither of them was modulated by TMS. Nevertheless, behavioural variables correlated with connectivity in the communities. The correlations were replicated in the two experiments, demonstrating that the changes in the analysed network dynamics at the relevant time window were induced by TMS.

Methodological limitations have largely constrained the quantification of neural dynamics of threat processing at high temporal resolution, harshening the delivery of personalized non-invasive and detailed descriptions of this particular behaviour in humans. The exact characterization of these neurobiological processes is, however, essential for identifying individuals at higher risk of affective or stress-related mental disorders. In so, a current limitation is that despite our results that evidenced that the interplay between synchrony of oscillations and network architecture is key in mediating efficient information transfer, an open question remains regarding long-term effects, as well as their particular alterations in neuropsychiatric disorders. However, we provide compelling evidence of how brain networks re-organize during physiological threat processing by establishing a framework that can be easily translated into studies including patient populations. In fact, the involvement of the CEN, SN and DMN during the threat processing gains importance, since they are not only key for high-order cognitive functions but further have a central role in long-term neuropsychiatric outcomes.^70,90,91^ Within these networks, CEN showed patterns of connectivity and information flow in the theta band that were not seen in alpha. As the role of theta and alpha oscillations during threat processing is increasingly recognized in humans,^17,19,92^ the connectivity alterations suggest that CEN plays a pivotal role in threat processing. A further limitation is that the selected time windows of 250 ms may to fully allow to elucidate the full dynamics of brain oscillatory activity, particularly in the alpha range. However, these time windows are within the range of those used in the literature.^93-95^ Further, brain oscillations can vary across individuals, and this may be influenced by several factors, including sex, which was not evaluated in the current study. Finally, our experimental settings did not allow to test TMS-related changes in behaviour, and, thus, its relevance remains to be elusive. Therefore, we encourage further research on this topic specifically looking for sex effects and behavioural outcomes.

Overall, our findings evidence that threat processing is related to changes in the brain’s modular architecture involving the DMN, SN and CEN. Changes in network topology in these three networks are a prerequisite for threat processing and related to behavioural responses. TMS modulation of theta and alpha oscillations changed the dynamics of network flexibility CEN, SN and DMN and information flow in SN and DMN. These observations suggest that this dynamical network re-organization during threat processing serve as efficient mechanisms for coping.

## Supplementary Material

fcad035_Supplementary_Data

## Abbreviations

AC =: accumbens
AG =: angular gyrus
AM =: amygdala
CA =: caudate
CC =: cuneal cortex
CEN =: central executive network
CGa =: cingulate gyrus anterior division
CGp =: cingulate gyrus posterior division
CO =: central opercular cortex
CS =: conditioned stimulus
DMN =: default mode network
dmPFC =: dorsomedial prefrontal cortex
EC =: effective connectivity
EEG =: electroencephalography
FMC =: frontal medial cortex
fMRI =: functional MRI
FO =: frontal orbital cortex
FOC =: frontal operculum cortex
FP =: frontal pole
HG =: Heschl’s gyrus
HI =: hippocampal region
IC =: insular cortex
ICC =: intracalcarine cortex
IFGo =: inferior frontal gyrus pars opercularis
IFGt =: inferior frontal gyrus pars triangularis
ITGa =: inferior temporal gyrus anterior division
ITGp =: inferior temporal gyrus posterior division
ITGto =: inferior temporal gyrus temporooccipital
L =: left hemisphere
LG =: lingual gyrus
LOi =: lateral occipital inferior division
LOs =: lateral occipital superior division
LPP =: late positive potential
LV =: lateral ventricle
MFG =: middle frontal gyrus
MTGa =: middle temporal gyrus anterior division
MTGp =: middle temporal gyrus posterior division
MTGto =: middle temporal gyrus temporooccipital
OFG =: occipital fusiform gyrus
OP =: occipital pole
PA =: pallidum
PC =: precuneus
PCG =: paracingulate gyrus
PGa =: parahippocampal gyrus anterior division
PGp =: parahippocampal gyrus posterior division
PoC =: post central gyrus
POC =: parietal operculum cortex
PP =: planum polare
PrC =: precentral gyrus
PT =: planum temporale
PU =: putamen
R =: right hemisphere
SC =: subcallosal cortex
SFG =: superior frontal gyrus
SMA =: supplementary motor area
SMGa =: supramarginal gyrus anterior division
SMGp =: supramarginal gyrus posterior division
SN =: salience network
SP =: supracalcarine cortex
SPL =: superior parietal lobule
STGa =: superior temporal gyrus anterior division
STGp =: superior temporal gyrus posterior division
TFCa =: temporal fusiform cortex anterior division
TFCp =: temporal fusiform cortex posterior division
TH =: thalamus
TMS =: transcranial magnetic stimulation
TOF =: temporal occipital fusiform cortex
TP =: temporal pole
TPDC =: temporal partial directed coherence
US =: unconditioned stimulus
