# Correction to: Modular segregation drives causality of the dynamic oscillatory network responses during threat processing

**DOI:** 10.1093/braincomms/fcad157

**Published:** 2023-05-22

This is a correction to: Gabriel Gonzalez-Escamilla, Venkata C Chirumamilla, Nabin Koirala, Abdul R Anwar, Oliver Tüscher, Johannes Vogt, Phillip Horstmann, Benjamin Meyer, George A Bonanno, Sergiu Groppa, Muthuraman Muthuraman, Modular segregation drives causality of the dynamic oscillatory network responses during threat processing, *Brain Communications*, Volume 5, Issue 2, 2023, fcad035, https://doi.org/10.1093/braincomms/fcad035

In the originally published version of this manuscript, the affiliation of one of the authors, Prof. Johannes Vogt, was incorrect. The correct affiliation for Prof. Vogt is as follows:

Department of Molecular and Translational Neuroscience, Institute of Anatomy II, Cluster of Excellence-Cellular Stress Response in Aging-Associated Diseases (CECAD), Center of Molecular Medicine Cologne (CMMC), University of Cologne, Cologne, Germany

This error has been corrected online.

