# Supplementary material for: Modular segregation drives causality of the dynamic oscillatory network responses during threat processing
Source: Brain Commun. 2023 Feb 17;5(2):fcad035. doi: 10.1093/braincomms/fcad035 (PMC9989139; doi:10.1093/braincomms/fcad035)
Supplement: fcad035_Supplementary_Data [file fcad035_supplementary_data.pdf]

Community driven causality of the dynamic oscillatory network responses during threat processing

Supplementary Materials

Methods

MRI data acquisition

For all participants magnetic resonance images were acquired at the Neuroimaging Center (NIC) Mainz, Germany, using a 3 Tesla MRI scanner (Magnetom Tim Trio, Siemens Healthcare, Erlangen, Germany) equipped with a 32-channel head coil. A Magnetization-prepared rapid gradient-echo (MP-RAGE) sequence was utilized (Repetition Time [TR] = 1900ms; Echo Time [TE] = 2.54ms; Inversion Time [IT] = 900; Pixel Bandwidth = 180; Acquisition Matrix = 320, 320; Flip Angle = 9°; voxel size = 0.8125, 0.8125 mm; Slice Thickness = 0.8 mm) was used.

Heart rate estimation

In the EEG signals, volume conduction is thought to be linear and instantaneous, and it is expected that the sources of cardiac signals are not generally time locked to the sources of EEG activity, reflecting the activity of cortical neurons.[1] The lCA can accurately identify the time courses of activation and scalp topographies of relatively large and temporally-independent sources from simulated scalp recordings, even in the presence of a large number of low-level and temporally-independent source activities.[2]

For heart rate detection analysis, the rows of the input matrix y are the EEG signals recorded at the 256 electrodes, the rows of the output data matrix $\vartheta = \mathrm{X}\mathrm{y}$ are time courses of activation of the lCA components, and the columns of the inverse matrix, X^{-1}, give the projection strengths of the respective components onto the scalp sensors.

In general, and unlike PCA, the component time courses of activation will be non-orthogonal. Corrected EEG signals can then be derived as $\mathrm{y}' = (\mathrm{X})^{-1}\vartheta'$, where ϑ' is the matrix of activation waveforms,$\vartheta\, v$, with rows representing sources of cardiac artifacts which are then extracted for further estimations from each participant. In total for experiment 1 we concatenated the 36 CS+ trials to take a total of 180 seconds and 24 CS- trials to take 120 seconds. For experiment 2 we concatenated the 54 CS+ trials to take 270 seconds and 36 CS- trials to take 180 seconds.

Reliability check of the EEG signals using inter-trial phase coherence (ITPC) analyses

Single trial data were first decomposed into their time-frequency representation by using the multitaper method.[3, 4] In this method the spectrum is estimated by multiplying the data with K different windows (i.e. tapers). In this study, $\mathrm{K} = 7$ orthogonal tapers were used with good leakage and spectral properties, the discrete prolate spheroidal sequences (DPSS) are applied.[5] The electrodes were grouped in line with the five lobes to have a global robust measure over a

certain lobe instead of selecting certain electrodes. The ITPC was estimated in theta (4-7 Hz) and alpha (8-13 Hz) frequency bands separately. This measure is stimulus-locked and independent of amplitude changes. A value of 0 represents absence of synchronization and a value of 1 indicates perfect synchronization. The baseline activity was taken as a reference and was calculated as the average at each frequency band and across conditions, from –250 to 0 ms before visual stimuli. The change with respect to the baseline interval at 6 windows of 250 ms each after the visual stimulus was then extrapolated up to 1500 ms. Finally, the ITPC difference between the CS+ and CS- was estimated. The significance levels of the ITPC are assessed using surrogate data by randomly shuffling 1000 times the single-trial spectral estimates from different latency windows during the baseline period.

Reconstruction of brain activity

The forward problem is the computation of the scalp potentials for a set of neural current sources. An established procedure was used by estimating the lead-field matrix with specified models for the brain; a volume conduction model with a finite-element method (FEM) was used.[6] For the forward modelling the surfaces of the compartments like the skin, skull, CSF, gray matter, and white matter extracted from the individual T1 MRI, and individual electrode locations were used. The forward modeling and the source analysis were done in FieldTrip.[7] The lead-field matrix (LFM) contains information about the geometry and conductivity of the model. The complete description of the solution for the forward problem has been described previously.[8, 9] A full description of the beamformer linear constrained minimum variance spatial filter is given elsewhere.[10, 11] The output of the beamformer at a voxel in the brain can be defined as a weighted sum of the output of all EEG channels. The weights determine the spatial filtering characteristics of the beamformer and are selected to increase the sensitivity to signals from a voxel and reduce the contributions of signals from (noise) sources at different locations. The frequency components and their linear interaction are represented as a cross-spectral density (CSD) matrix. In order to visualize power at a given frequency range, a linear transformation was used based on a constrained optimization problem, which acts as a spatial filter.[10] The spatial filter assigned a specific value of power to each voxel. For a given source the beamformer weights for a location of interest are determined by the data covariance matrix and the LFM. A voxel size of 5 mm was used in this study, resulting in 6676 voxels covering the entire brain. The created source model was then interpolated on the brain regions defined according to the Harvard Oxford cortical-subcortical regions of interest (ROIs) defined in the MNI space. For each frequency band (theta and alpha) the activated voxels were selected by a

within-subject surrogate analysis to define the significance level, which was then used to identify voxels in the regions as activated voxels. Once the brain region voxels were identified, their activity was extracted from the source space. In a further analysis, all the original source signals for each Harvard-Oxford[12] region with several activated voxels were combined by estimating the second order spectra and employing a weighting scheme depending on the analyzed frequency range to form a pooled source signal estimate for each region as previously described separately for both stimulus (Cs+, CS-).[13-15] Finally, the time series difference between the two conditions (CS+, CS-) was obtained for all the following analyses.

Investigating causal relationships between network nodes

The effective connectivity analysis was performed on all the nodes separately, for each of the three new communities and each frequency separately. Using time-frequency causality, is possible not only to focus on a particular frequency, but also to analyze the dynamics of the causality at that frequency. The time-frequency causality estimation using the TPDC is based on dual extended Kalman filtering (DEKF),[16, 17] and allows time-dependent auto regressive (AR) coefficients to be estimated. One EKF estimates the states and feeds this information to the other; the second EKF estimates the model parameters and shares this information with the first. By using two Kalman filters working in parallel with one another, we can estimate both states and model parameters of the system at each time instant. After estimating the time-dependent multivariate (MVAR) coefficients, the next step is to use those coefficients for the calculation of causality between the time series. By calculating the time-dependent MVAR coefficients at each time point, we can also calculate partial directed coherence (PDC) at each time point. The frequency bands taken into account were the theta and alpha. After estimating the TPDC values the significance level was calculated from the applied data using a bootstrapping method [18]. In short, we divide the original time series into smaller non-overlapping windows and randomly shuffle the order of these windows to create a new time series. Data length was taken from -250 milliseconds to 1500 milliseconds i.e. 1750 milliseconds. Number of data points was computed by taking the product of the data length and sampling rate (1.750secs X 250 = 437.5 data points). Data length was at least 438 data points (multiplied with number of signals) for sub-samples. Model order used was 30 and data length was long enough for this model order. The model order was estimated for the first sub-interval and then it was fixed for the subsequent samples. We used Akaike's final perdition error as our model estimation criterion. Based on the literature, the model order can be estimated using Schwarz Bayesian Criterion (SBC) or Akaike's final prediction error (FPE). However, the FPE

tends to over-estimate the model order than SBC because SBC favors space solutions and penalizes the number of parameters more strongly than FPE.[19] Moreover, it is also generally preferred to select model order that is slightly larger large than the other way around.[19] Hence, we used Akaike's model. To note, it has been shown that a small variation of model order do not significantly alter the connectivity patterns.[20] Time-varying MVAR coefficients were calculated using sub-intervals so that the dynamics of MVAR coefficients can be captured. The MVAR model is fitted to the shuffled time series and TPDC is estimated. The bootstrapping is performed 1000 times and the average TPDC value is taken as the significance threshold for all our connections. This process is performed separately for each participant. The resulting value is the significance threshold value for all our connections. This process is performed separately for each subject. In this study the open source Matlab package autoregressive fit (ARFIT)[21, 22] was used for estimating the autoregressive coefficients from the spatially filtered source signals of the identified nodes in the three new communities. We applied time reversal technique TRT[23] as a second significance test on the connections already identified by TPDC using a data-driven bootstrapping surrogate significance test.

Results

Threat processing related inter-trial phase coherence changes

The frontal theta showed a significant inter-trial phase coherence (ITPC) increase in between the baseline (-250-0 ms) and the four subsequent time windows from (0 to 1000 ms) (experiment 1 $p < 0.001$; experiment 2 $p < 0.001$; supplementary Fig. 1). The occipital alpha showed decreased ITPC between the two conditions (CS+ and CS-) but the difference was only significant between the baseline and the subsequent three time windows from (T1 to T3, 0-750 ms) in both experiment 1 ($p < 0.001$) and experiment 2 ($p < 0.001$). The ITPC in the theta frequency showed an inverted U-shape like temporal pattern, increase in the first two windows (T1 = 0-250 and T2 = 250-500 ms) and then reduced in the two subsequent time windows (T3 = 500-750 and T4 = 750-1000 ms) in both experiments. The increase in ITPC of the frontal lobe showed the robust nature of the oscillatory response of each trial for the threat processing stimuli. The occipital alpha showed the opposite behavior, a decrease in the first two windows (T1 and T2) after the visual stimuli followed by an increase in the time windows T3 and T4 also in both experiments. The decrease of ITPC in the occipital lobe showed the specificity of attention for threat stimuli.

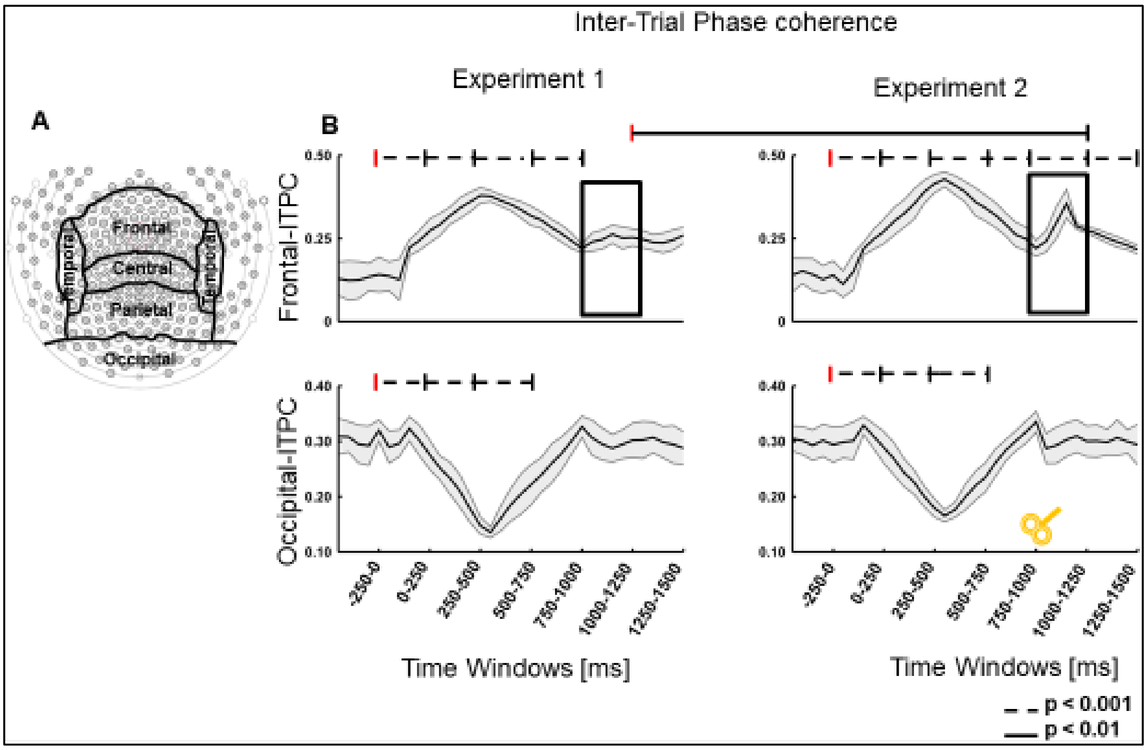

Supplementary Fig. 1: inter-trial phase coherence. A) Shows the subdivision of the lobes for the estimation of the inter-trial phase coherence. B) First row shows the frontal inter-trial phase coherence (ITPC) for both the experiments and time windows, starting from the baseline (-250 to 0 milliseconds [ms]) followed by six time windows (T1-T6) each 250 ms (frontal experiment 1 $p < 0.001$; experiment 2 $p < 0.001$), the second row shows the occipital ITPC (experiment 1 $p < 0.001$; experiment 2 $p < 0.001$). The dashed black line indicates the significant differences and the red vertical line indicates the window to which the comparison was done. The black boxes in B) indicate the change in ITPC between the experiment 1 and experiment 2. The TMS coil indicates the time of application of single pulse TMS. Reported statistical values for significant differences between the time intervals were obtained from a two-way factorial ANOVA, all the intervals were also compared to the baseline.

TMS induced inter-trial phase coherence changes

In experiment 2, the ITPC at the theta band was perturbed by the TMS in the dmPFC at 1000 ms and induced a significant increase ($p < 0.01$) in the frontal theta but did not affect the occipital alpha (supplementary Fig. 1). We were able to modulate the ITPC using TMS at the dmPFC in the frontal lobe during the time interval of threat processing. The control experiment of TMS at 80 ms did not induce any significant change in the frontal lobe indicating choosing the correct temporal window for perturbation is vital.

Topological characteristics of dynamic community alliance in the alpha band

In central executive network (CEN), the alpha band did not exhibit any significant differences in neither experiment nor analyzed network measures.

For the salience network (SN) (Supp. Fig. 2) no significant decreases with respect to baseline were detected for T1 to T4 in neither experiment. However, flexibility significantly decreased between T5 and T4 in both experiments ($p < 0.001$). The clustering coefficient also showed no changes in respect to baseline in neither experiment. However, a decrease in clustering coefficient in T5 in comparison to T4 was significant in both experiments ($p < 0.001$). For local and global efficiency, no significant changes with respect to baseline were detected in neither experiment. The decrease in global efficiency in T5 compared to T4 was significant in both experiments ($p < 0.001$). The decrease in local efficiency in T5 compared to T4 was significant in both experiments ($p < 0.001$).

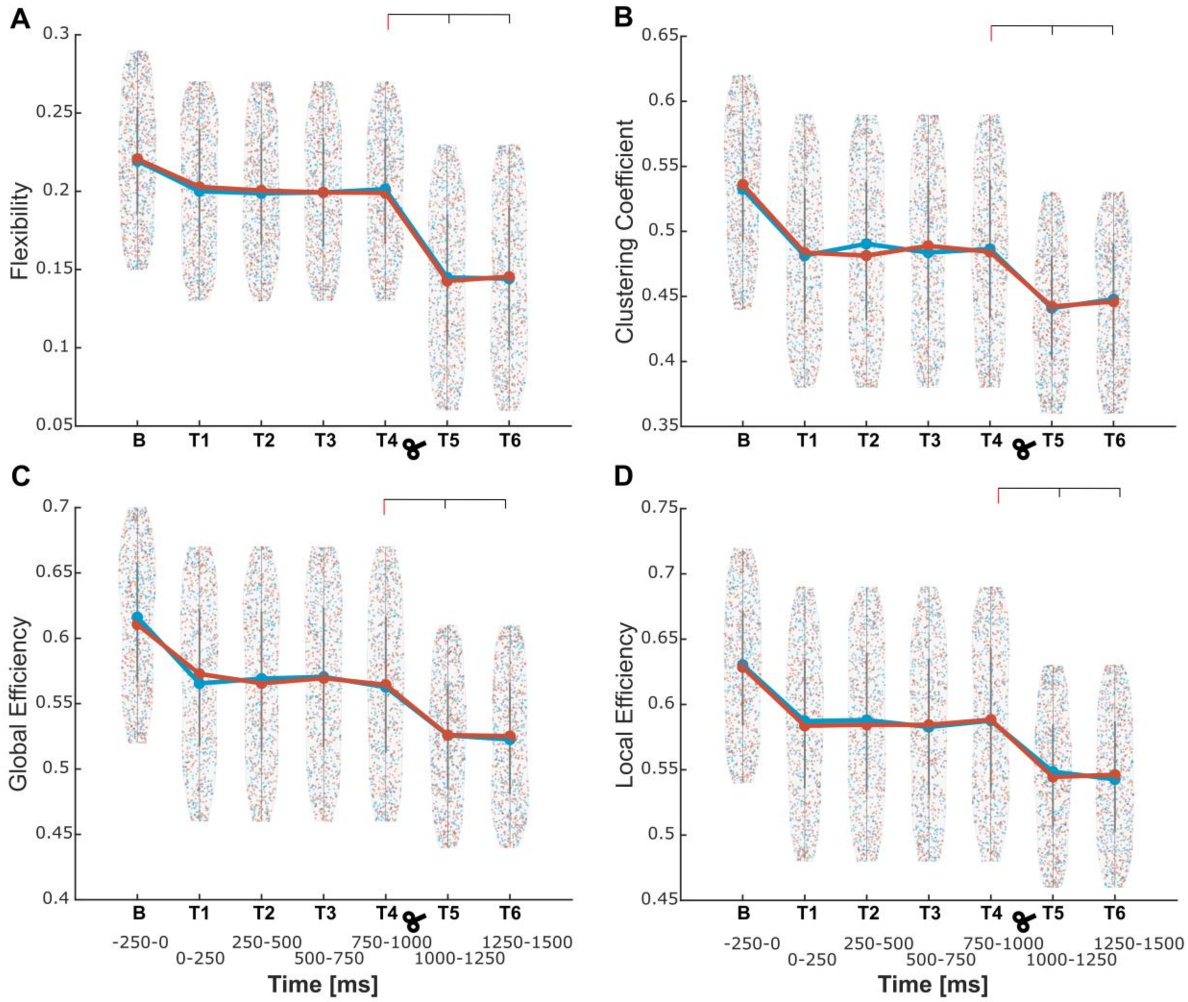

Supplementary Fig. 2. Topological dynamic characteristics for the Salience Network (SN) in the alpha frequency band. A) The network flexibility starting from the baseline (-250 to 0) milliseconds (ms) window to all the following six-time windows (T1-T6) for every 250 ms separately with mean and distribution (experiment 1, factor condition ($F_{1,18} = 6.47$, $p = 0.015$) and time ($F_{6,108} = 4.54$, $p = 0.007$) and in experiment 2, factor condition ($F_{1,25} = 5.68$, $p = 0.025$) and time ($F_{6,150} = 3.90$, $p = 0.015$)). B), C), and D) show the values for, clustering coefficient (experiment 1, factor condition ($F_{1,18} = 0.96$, $p = 0.67$) and time ($F_{6,108} = 1.26$, $p = 0.36$) and experiment 2, factor condition ($F_{1,25} = 1.45$, $p = 0.51$) and time ($F_{6,150} = 0.69$, $p = 0.28$)), global efficiency (experiment 1, factor condition ($F_{1,18} = 0.57$, $p = 0.85$) and time ($F_{6,108} = 0.93$, $p = 0.64$) and experiment 2, factor condition ($F_{1,25} = 1.32$,

p = 0.31) and time ($F_{6,150}$ = 1.24, p = 0.51)), and local efficiency (experiment 1, factor condition ($F_{1,18}$ = 7.67, p = 0.007) and time ($F_{6,108}$ = 4.48, p = 0.013) and experiment 2, factor condition ($F_{1,25}$ = 11.45, p = 0.005) and time ($F_{6,150}$ = 5.68, p = 0.005)), respectively. Density plots are presented with data points from *Experiment 1* (without TMS) in blue and *Experiment 2* (with TMS) in red obtained for each density and time windows. The mean value across all data points is depicted with the larger circle for each experiment, while the bars indicate the standard deviation. For each parameter and experiment, the points depict values at each network density for each participant and each of the 20 densities. Reported F and p values for significant differences between the time intervals were obtained from a two-way factorial ANOVA, all the intervals were also compared to the baseline for both experiments.

In the alpha band, the DMN (Supp. Fig 3) showed decreased flexibility in experiment 1 (factors condition ($F_{1,18}$ = 8.48, p = 0.01) and time ($F_{6,108}$ = 3.68, p = 0.009)), but increased flexibility in experiment 2 (factors condition ($F_{1,25}$ = 5.45, p = 0.03) and time ($F_{6,150}$ = 2.86, p = 0.01)). Despite these contrasting findings, flexibility was increased T5 compared to T4 in both experiments (all $p < 0.001$). In the alpha band, clustering coefficient was decreased in both experiments (experiment 1, factors condition ($F_{1,18}$ = 7.25, p = 0.01) and time ($F_{6,108}$ = 4.57, p = 0.007); experiment 2, factors condition ($F_{1,25}$ = 4.35, p = 0.01) and time ($F_{6,150}$ = 2.98, p = 0.009)). In both experiments, increase clustering coefficients was observed for T5 compared to T4 (all $p < 0.001$).
DMN alpha band, global and local efficiency was decreased in both experiments (experiment 1, factors condition ($F_{1,18}$ = 6.48, p = 0.008; $F_{1,18}$ = 4.26, p = 0.026) and time ($F_{6,108}$ = 5.87, p = 0.005; $F_{6,108}$ = 3.16, p = 0.014); experiment 2 (factors condition ($F_{1,25}$ = 3.25, p = 0.02; $F_{1,25}$ = 4.26, p = 0.022) and time ($F_{6,150}$ = 3.78, p = 0.005; $F_{6,150}$ = 5.46, p = 0.009)). An increase of clustering coefficient and global efficiency was observed for T5 compared to T4 in both experiments ($p < 0.001$).

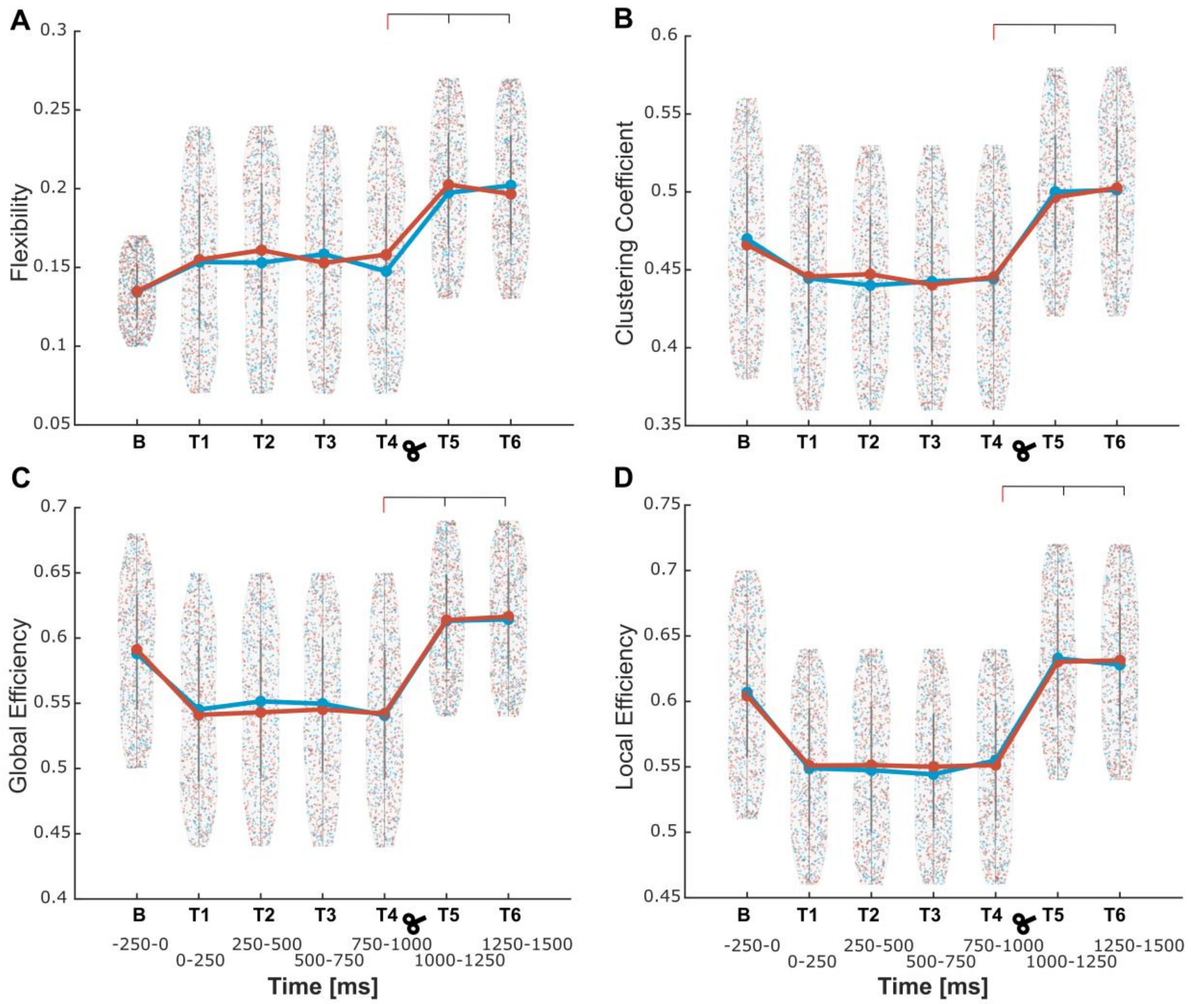

Supplementary Fig. 3. Topological dynamic characteristics for the Default Mode Network (DMN) in the alpha frequency band. A) The network flexibility starting from the baseline (-250 to 0) milliseconds (ms) window to all the following six-time windows (T1-T6) for every 250 ms separately with mean and distribution (experiment 1, factors condition ($F_{1,18}$ = 8.48, p = 0.01) and time ($F_{6,108}$ = 3.68, p = 0.009); experiment 2, factors condition ($F_{1,25}$ = 5.45, p = 0.03) and time ($F_{6,150}$ = 2.86, p = 0.01)). B), C), and D) show the values for clustering coefficient (experiment 1, factors condition ($F_{1,18}$ = 7.25, p = 0.01) and time ($F_{6,108}$ = 4.57, p = 0.007); experiment 2, factors condition ($F_{1,25}$ = 4.35, p = 0.01) and time ($F_{6,150}$ = 2.98, p = 0.009)), global efficiency (experiment 1, factors condition ($F_{1,18}$ = 6.48, p = 0.008) and time ($F_{6,108}$ = 5.87, p = 0.005); experiment 2 (factors condition ($F_{1,25}$ = 3.25, p = 0.02) and time ($F_{6,150}$ = 3.78, p = 0.005;)), and local efficiency (experiment 1, factors condition ($F_{1,18}$ = 4.26, p = 0.026) and time ($F_{6,108}$ = 3.16, p = 0.014); experiment 2 (factors condition ($F_{1,25}$ = 4.26, p = 0.022) and time ($F_{6,150}$ = 5.46, p = 0.009)), respectively. Density plots are presented with data points from *Experiment 1* (without TMS) in blue and *Experiment 2* (with TMS) in red obtained for each density and time windows. The mean value across all data points is depicted with the larger circle for each experiment, while the bars indicate the standard deviation. For each parameter and experiment, the points depict values at each network density for each participant and each of the 20 densities. Reported F and p values for significant differences between the time intervals were obtained from a two-way factorial ANOVA, all the intervals were also compared to the baseline for both experiments.

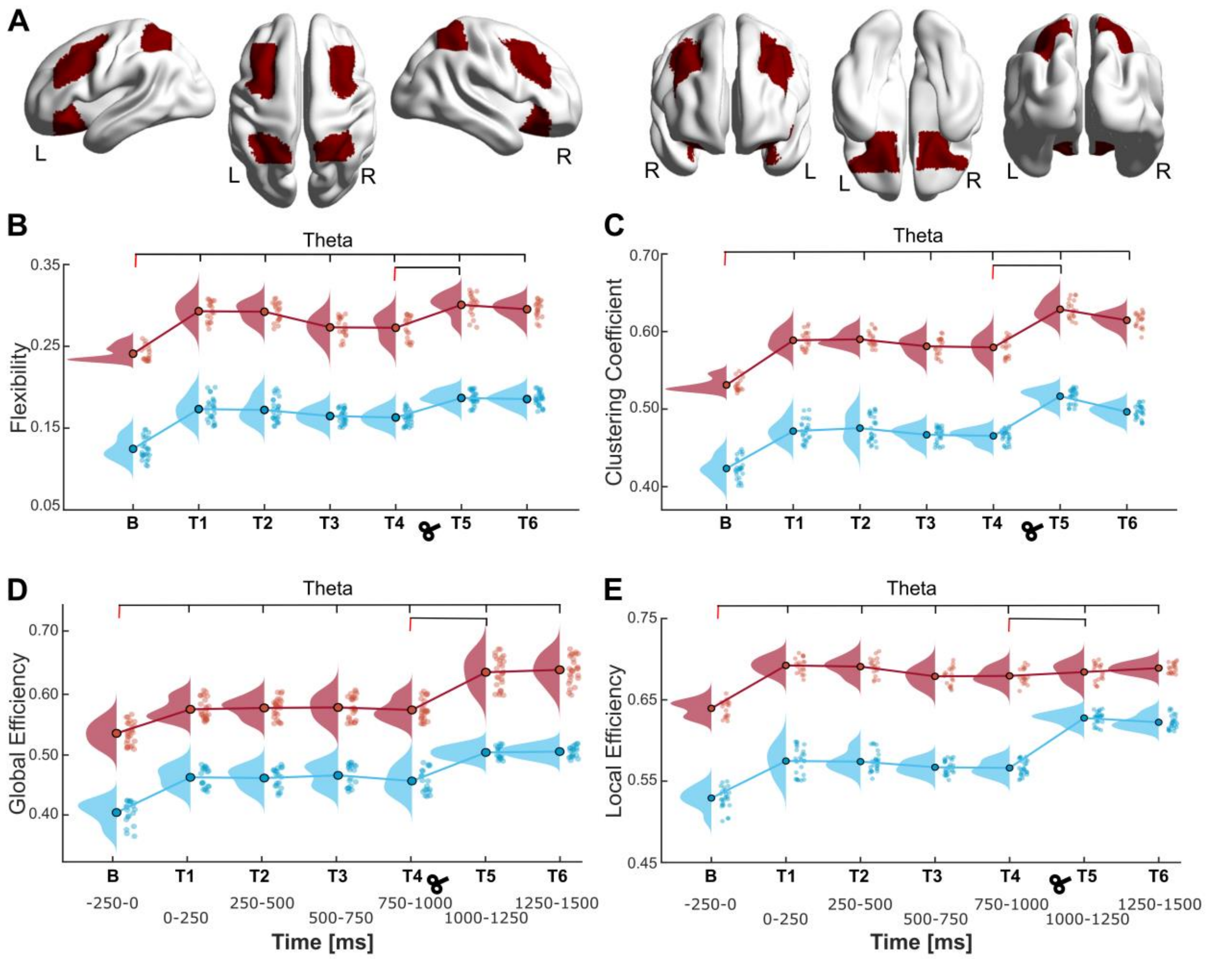

Supplementary Fig. 4. Topological dynamic characteristics within the central executive network (CEN) in the theta frequency. A) Depiction of the regions conforming the CEN during threat processing, the corresponding region list is given in Table 1, marked in red color. B) The network flexibility is shown starting from the baseline (B, -250 to 0 ms) window to all the following six time windows (T1-T6, every 250 milliseconds [ms]) separately (experiment 1, factor condition ($F_{1,18}$ = 20.43, $p < 0.001$) and factor time ($F_{6,108}$ = 10.35, $p < 0.001$); and in experiment 2, factor condition ($F_{1,25}$ = 22.36, $p < 0.001$) and time ($F_{6,150}$ = 13.24, $p < 0.001$)). C), D), and E) depict the same as B) but for the clustering coefficient (experiment 1, factor condition ($F_{1,18}$ = 35.21, $p < 0.001$) and time ($F_{6,108}$ = 17.45, $p < 0.001$) and experiment 2, factor condition ($F_{1,25}$ = 30.11, $p < 0.001$) and time ($F_{6,150}$ = 14.89, $p < 0.001$)), global efficiency (experiment 1, factor condition ($F_{1,18}$ = 9.30, $p = 0.004$) and time ($F_{6,108}$ = 6.69, $p < 0.001$); experiment 2, factor condition ($F_{1,25}$ = 7.53, $p < 0.001$) and time ($F_{6,150}$ = 6.93, $p < 0.001$)), and local efficiency (experiment 1, factor condition ($F_{1,18}$ = 38.94, $p < 0.001$) and time ($F_{6,108}$ = 19.68, $p < 0.001$) and experiment 2, factor condition ($F_{1,25}$ = 35.86, $p < 0.001$) and time ($F_{6,150}$ = 17.29, $p < 0.001$)), respectively. Violin plots are presented with data points (mean of each participant) for Experiment 1 (without TMS) in light blue and Experiment 2 (with TMS) in red obtained for each density and time windows. The mean value across participants is depicted with the bold lined circle with the same color for each experiment. Significant differences between the time intervals were attested using a two-way factorial ANOVA, all the intervals were also compared to the baseline for both experiments.

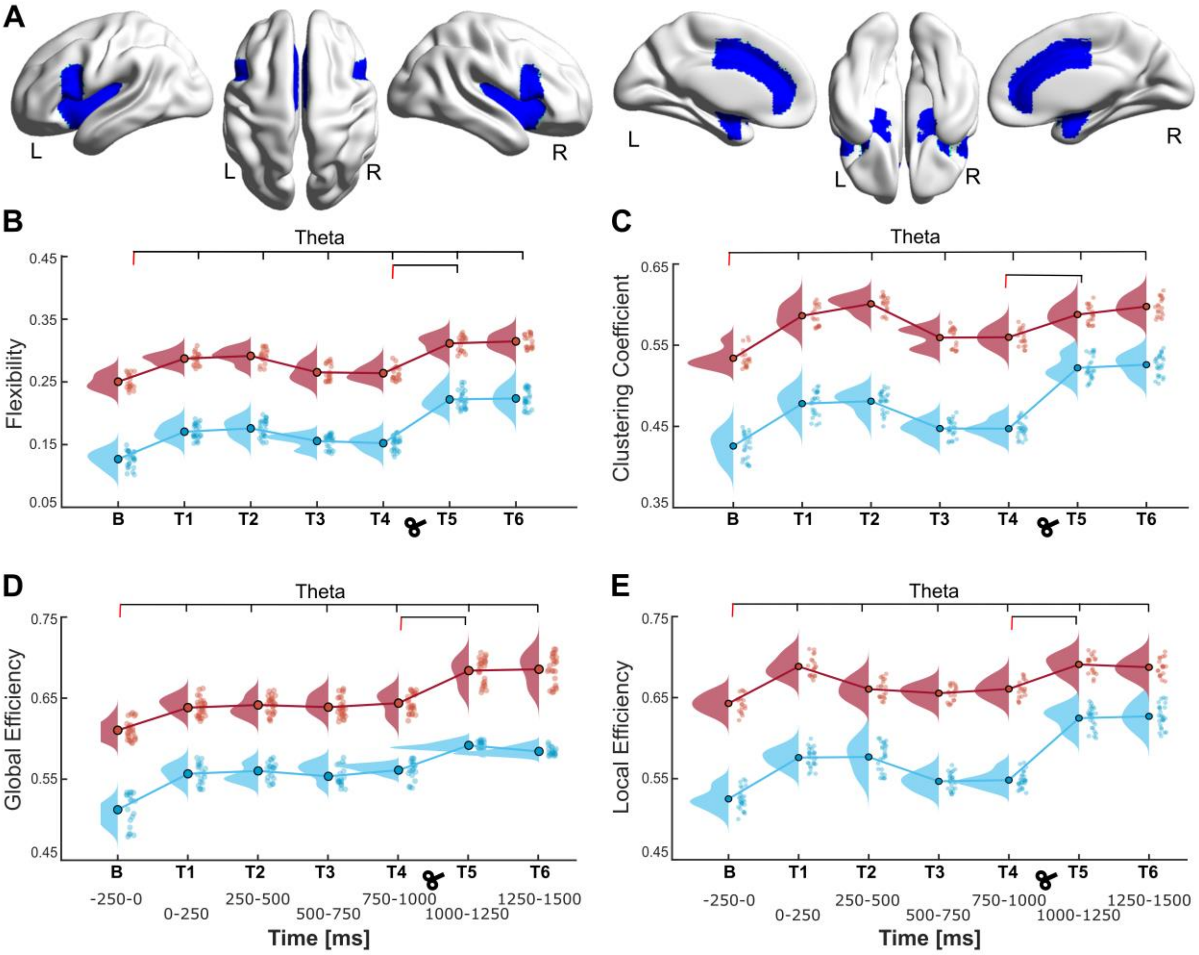

Supplementary Fig. 5. Topological dynamic characteristics within the salience network (SN) in the theta frequency. A) Depiction of the regions conforming the SN, the corresponding region list is given in Table 1, marked in blue color. B) The network flexibility is shown starting from the baseline (B, -250 to 0 milliseconds [ms]) window to all the following six time windows (T1-T6, every 250 ms) separately (experiment 1, factor condition ($F_{1,18} = 10.23$, $p = 0.005$) and time ($F_{6,108} = 5.12$, $p = 0.002$) and in experiment 2, factor condition ($F_{1,25} = 11.21$, $p = 0.008$) and time ($F_{6,150} = 6.79$, $p < 0.001$)). C), D), and E) depict the same as B) but for the clustering coefficient (experiment 1, factor condition ($F_{1,18} = 12.67$, $p = 0.008$) and time ($F_{6,108} = 4.78$, $p = 0.006$) and experiment 2, factor condition ($F_{1,25} = 10.21$, $p = 0.005$) and time ($F_{6,150} = 4.98$, $p = 0.008$)), global efficiency (experiment 1, factor condition ($F_{1,18} = 8.25$, $p = 0.004$) and time ($F_{6,108} = 6.87$, $p < 0.001$); experiment 2, factor condition ($F_{1,25} = 6.14$, $p = 0.002$) and time ($F_{6,150} = 7.89$, $p < 0.001$)), and local efficiency (experiment 1, factor condition ($F_{1,18} = 11.48$, $p = 0.002$) and time ($F_{6,108} = 3.95$, $p < 0.001$) and experiment 2, factor condition ($F_{1,25} = 10.69$, $p = 0.004$) and time ($F_{6,150} = 6.12$, $p < 0.001$)), respectively. Violin plots are presented with data points (mean of each participant) for Experiment 1 (without TMS) in light blue and Experiment 2 (with TMS) in red obtained for each density and time windows. The mean value across participants is depicted with the bold lined circle with the same color for each experiment. Reported F and p values for significant differences between the time intervals were obtained from a two-way factorial ANOVA, all the intervals were also compared to the baseline for both experiments.

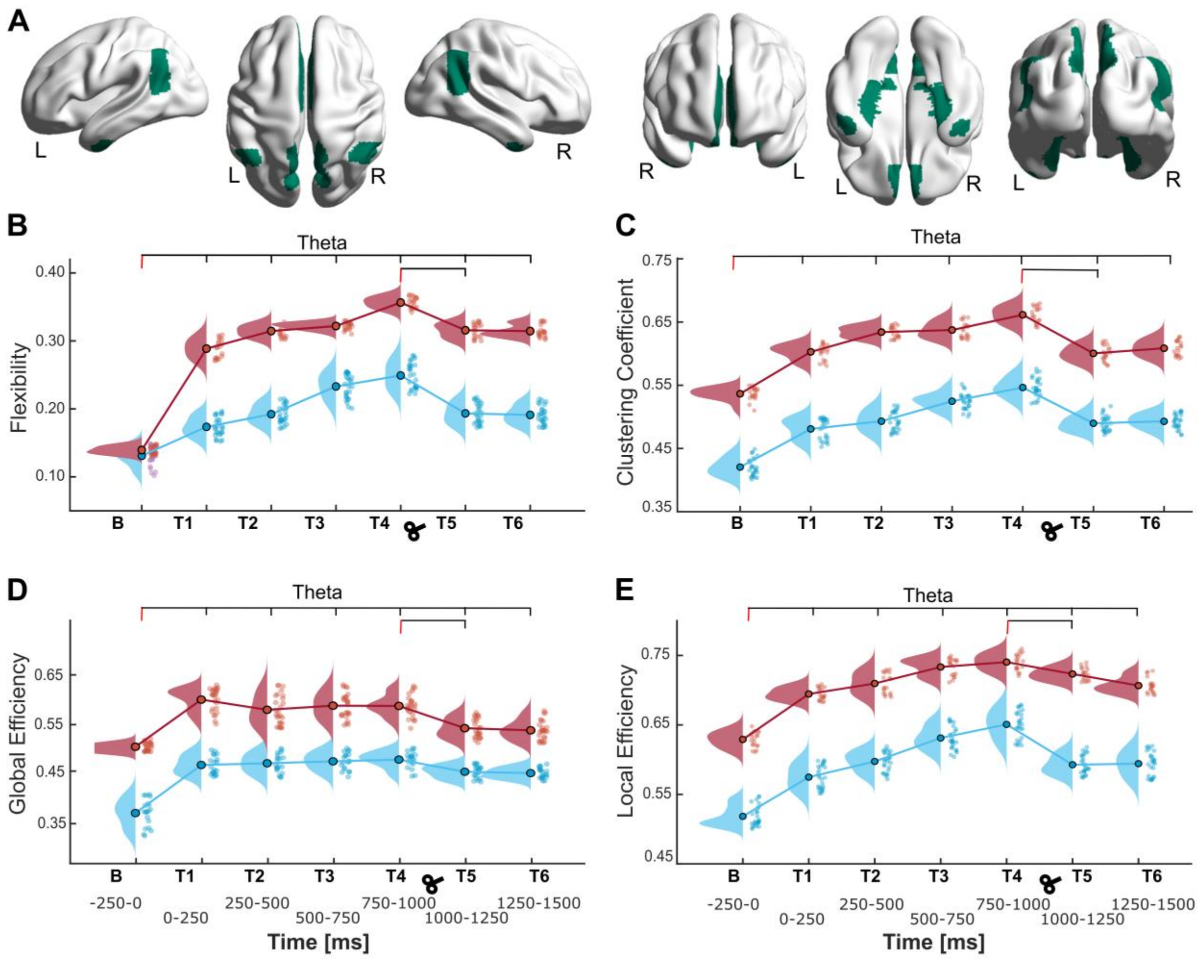

Supplementary Fig. 6. Topological dynamic characteristics within the default mode network (DMN) in the theta frequency. In A) the representative figure with regions comprised in the DMN, the corresponding list is given in Table 1, marked in green color. B) The network flexibility is shown starting from the baseline (B, -250 to 0 milliseconds [ms]) window to all the following six time windows (T1-T6, every 250 ms) separately (experiment 1, factor condition ($F_{1,18}$ = 17.90, p = 0.004) and time ($F_{6,108}$ = 8.90, p < 0.001) and experiment 2, the factor condition ($F_{1,25}$ = 18.67, p < 0.001) and time ($F_{6,150}$ = 7.83, p < 0.001)). C), D), and E) depict the same as B) but for clustering coefficient (experiment 1, factor condition ($F_{1,18}$ = 18.43, p < 0.001) and time ($F_{6,108}$ = 10.39, p < 0.001) and experiment 2, factor condition ($F_{1,25}$ = 19.59, p < 0.001) and time ($F_{6,150}$ = 8.90, p < 0.001)), global efficiency (experiment 1, factors condition ($F_{1,18}$ = 12.45, p < 0.001) and time ($F_{6,108}$ = 6.98, p < 0.001); experiment 2, factors condition ($F_{1,25}$ = 9.34, p < 0.001) and time ($F_{6,150}$ = 12.89, p < 0.001)), and local efficiency (experiment 1, factor condition ($F_{1,18}$ = 11.68, p = 0.001) and time ($F_{6,108}$ = 10.20, p < 0.001) and experiment 2, the factor condition ($F_{1,25}$ = 11.39, p < 0.001) and time ($F_{6,150}$ = 10.12, p < 0.001)), respectively. Violin plots are presented with data points (mean of each participant) for Experiment 1 (without TMS) in light blue and Experiment 2 (with TMS) in red obtained for each density and time windows. The mean value across participants is depicted with the bold lined circle with the same color for each experiment. Reported F and p values for significant differences between the time intervals were obtained from a two-way factorial ANOVA, all the intervals were also compared to the baseline for both experiments.

Control experiment 3 with TMS at 80 milliseconds

The three network parameters were estimated for the control experiment as described in the main manuscript. The parameters did not differ significantly ($p > 0.05$ for all three parameters between experiment 1 (without TMS) and experiment 3 (with TMS at 80 ms) (Supplementary Fig. 7). However, the T5 window (1000-1250 ms) differed significantly between the experiment 2 (with TMS) and experiment 3 for all the three parameters ($p < 0.01$) (refer to Supplementary Fig. 1). The control experiment results indicated that the TMS at 80 ms did not change the course of the dynamic network adaptation. On the other hand, this experiment validates that the correct temporal window for TMS is 1000 ms to modulate the network response in each of the three newly formed communities.

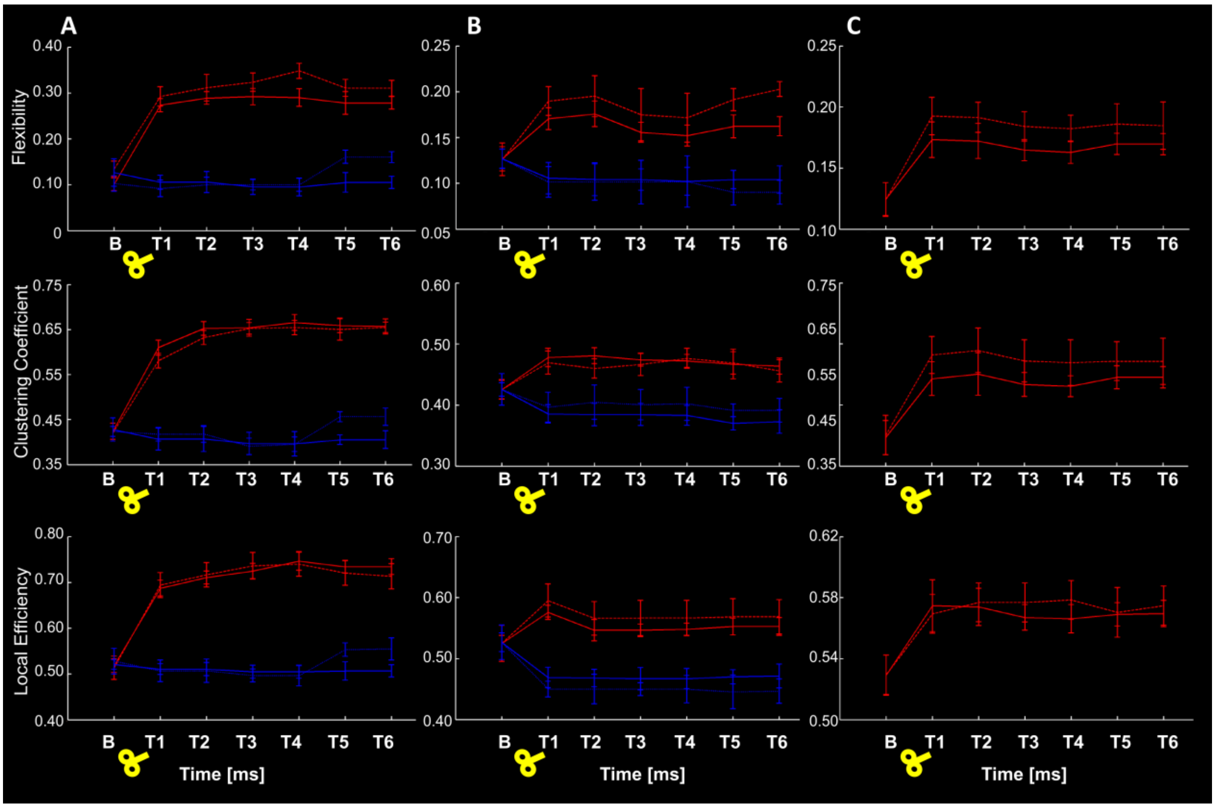

Supplementary Fig. 7. Network analysis results for the control experiment applying TMS at 80 milliseconds. A) Represent the DMN for the three network parameters flexibility, clustering coefficient and local efficiency. B) Represent the SN for the three network parameters flexibility, clustering coefficient and local efficiency. C) Third column represent the CEN for the three network parameters flexibility, clustering coefficient and local efficiency. The red lines indicate the mean and the standard deviation for the theta frequency band and the blue lines for the alpha frequency band. The dashed line represents the experiment 1 and the bold line represents the experiment 2 in all the three graphs A, B and C. The TMS coil indicates the time of application of single pulse TMS at 80ms.

Discussion

Inter-trial coherence as a substrate of threat processing

We found an increase of the theta inter-trial phase coherence in the frontal lobe and a

simultaneous decrease in the inter-trial phase coherence for the occipital alpha, relative to stimuli presentation. This suggests that threat processing is not a purely autonomous response to stimulus presentation, but rather that it facilitates interactions between regions[24, 25] and for specific temporal conditioned stimuli.[26] Previous studies on memory function have shown that there is a relationship between brain response to external stressors and the phase of the synchronized oscillations,[27, 28] which can be prolonged by exciting a small number of neurons[29] that participate in such oscillatory behavior. Low-frequency oscillations, such as theta (4–7 Hz) and alpha (8–12 Hz) can be recorded in different specific anatomical regions and especially facilitate communication between hippocampus,[30] amygdala,[31] and prefrontal cortex. The specificity of attention in instructed threat studies suggests that these oscillations provide a temporal window for inter-regional communication.[32] Intra-regional functional communication has been found for interactions involving the fronto-occipital circuit during directed attention to visual stimuli.[33, 34] It has also previously been shown that frontal theta phase consistency reflects coordination of information transfer between distant brain areas.[35, 36] On the contrary, the decrease in the inter-trial phase for the alpha oscillations in the occipital lobe could be related to alterations due to pre-visual threat processing. Earlier studies[37, 38] have shown disruption in phase consistency over successive trials in the occipital lobe, which suggest that the inter-trial coherence of these oscillations drives the physiological response during instructed threat processing. Our data support this hypothesis and localize it differentially in both the frontal and occipital lobes. Here we use the dynamics of theta driven alterations for the application of TMS pulses to dmPFC, and apply TMS at a time relevant for threat processing (1000 ms) and a time point before this (80 ms). Using this approach, we were able to achieve different effects at the network behavior level. Specifically, a TMS pulse before active processing leads to a community independent increase of network flexibility and clustering without a preservation of inter-network interactions. A TMS pulse applied during a time point physiologically relevant for processing mirrors the network response and intercommunity information transfer.

Validation using different brain atlas

To evaluate if the results obtained in the study are dependent on the choice of the brain atlas, we performed the community analysis and computed a network measure - Flexibility using a brain atlas with 100 regions of interests[39] mapped to seven functional networks.[40] We showed that the results are replicable (suppemmentary Fig. 8) for three functional networks dorsal attention, salience ventral attention and default mode corresponding to the ones we used in the

study - central executive, salience and default mode network. There was a significant increase in flexibility for the three networks respectively compared to baseline all the other following windows (factors condition ($F_{1,18} = 8.48$, $p = 0.01$; $F_{1,18} = 10.15$, $p < 0.001$; $F_{1,18} = 9.28$, $p < 0.001$) and time ($F_{6,108} = 3.68$, $p = 0.009$; $F_{6,108} = 5.09$, $p = 0.004$; $F_{6,108} = 6.42$, $p = 0.001$)), but increased flexibility in experiment 2 (factors condition ($F_{1,25} = 5.45$, $p = 0.03$; $F_{1,25} = 5.47$, $p = 0.004$; $F_{1,25} = 5.20$, $p = 0.003$) and time ($F_{6,150} = 2.86$, $p = 0.01$; $F_{6,150} = 4.89$, $p = 0.007$; $F_{6,150} = 6.29$, $p = 0.003$)). We also found, replicating our findings, a significant increase in the flexibility for the windows at T5 compared to T4 in both experiments ($p < 0.001$) for the two networks dorsal attention and salience ventral attention. In the default mode network, the flexibility decreased as in our original analyses for the theta frequency band between the windows comparing T5 and T4 for both experiments ($p < 0.001$). From these analyses, it is clear the network architecture shows similar dynamic processing (Flexibility) irrespective of the used atlas in this study.

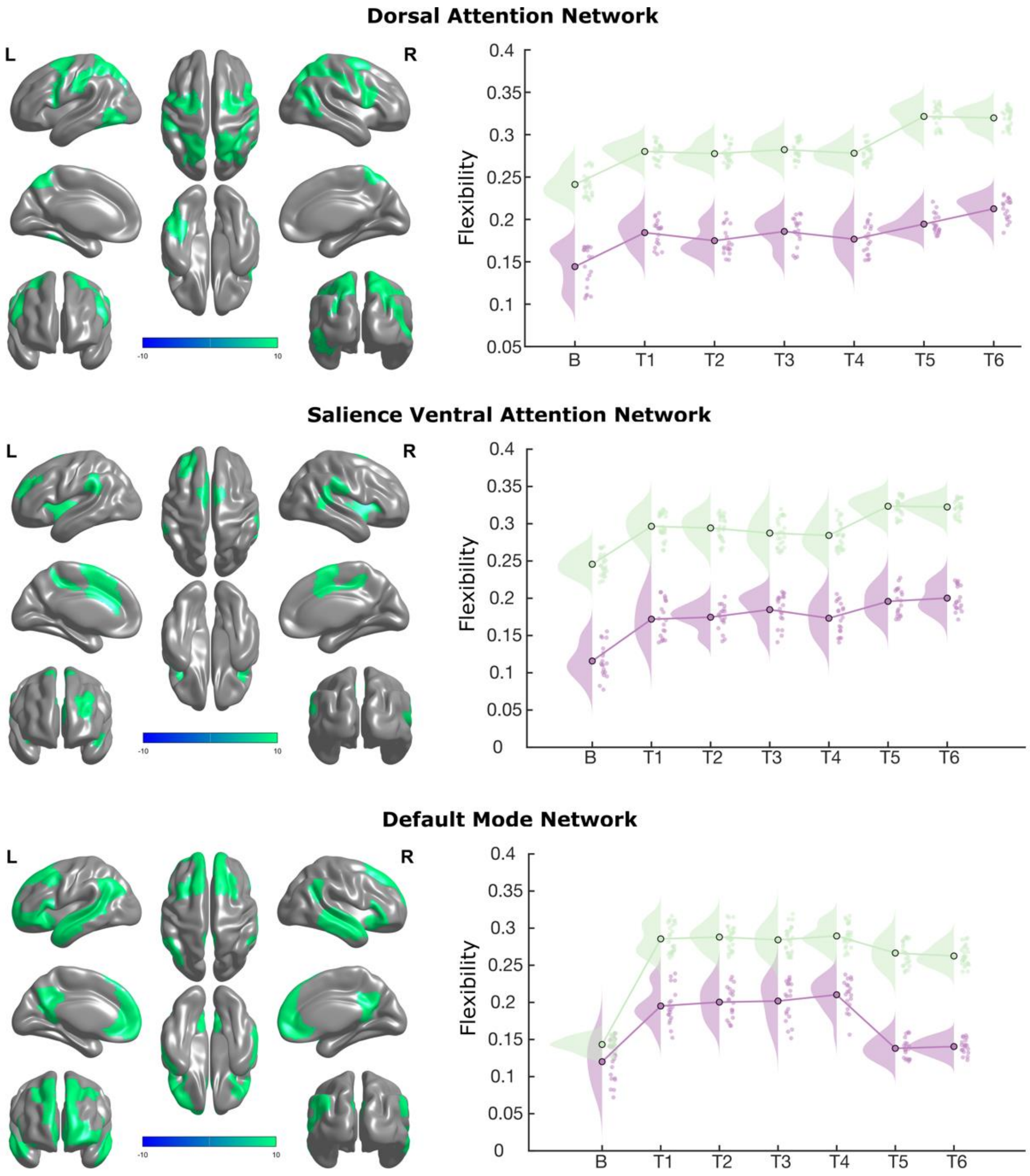

Supplementary Fig. 8. Depiction of the regions conforming the DAN, SVN and DMN during threat processing marked in green color, the atlas consisted on 100 regions of interest[39] that were mapped to seven functional networks.[40] The left column depicts the network flexibility starting from the baseline (B; -250 to 0 milliseconds [ms]) window to all the following six-time windows (T1-T6, every 250 ms). L: left hemisphere; R: right hemisphere.

References

1. Milad MR, Rauch SL, Pitman RK, Quirk GJ. Fear extinction in rats: implications for human brain imaging and anxiety disorders. *Biol Psychol*. 2006 2006;73(1):61-71.
2. Zhou W, Zhou J, Zhao H, Ju L. Removing eye movement and power line artifacts from the EEG based on ICA. *Conference proceedings : Annual International Conference of the IEEE Engineering in Medicine and Biology Society IEEE Engineering in Medicine and Biology Society Annual Conference*. 2005;2005:6017-20. doi:10.1109/IEMBS.2005.1615863
3. Mitra PP, Pesaran B. Analysis of dynamic brain imaging data. *Biophysical Journal*. 1999 1999;76(2):691-708.
4. Muthuraman M, Galka A, Deuschl G, Heute U, Raethjen J. Dynamical correlation of non-stationary signals in time domain--A comparative study. *Biomedical Signal Processing and Control*. 2010 2010;5(3):205-213.
5. Pollak HO, Slepian D. Prolate spheroidal wave functions, Fourier analysis and uncertainty, I. *Bell system Technical Journal*. 1961 1961;40:43-64.
6. Wolters CH, Anwander A, Berti G, Hartmann U. Geometry-adapted hexahedral meshes improve accuracy of finite-element-method-based EEG source analysis. *IEEE transactions on bio-medical engineering*. 2007 2007;54(8):1446-53.
7. Oostenveld R, Fries P, Maris E, Schoffelen JM. FieldTrip: Open source software for advanced analysis of MEG, EEG, and invasive electrophysiological data. *Computational intelligence and neuroscience*. 2011;2011:156869. doi:10.1155/2011/156869
8. Muthuraman M, Heute U, Deuschl G, Raethjen J. The central oscillatory network of essential tremor. *Conf Proc IEEE Eng Med Biol Soc*. 2010 2010;1:154-7.
9. Muthuraman M, Heute U, Arning K, et al. Oscillating central motor networks in pathological tremors and voluntary movements. What makes the difference? *Neuroimage*. 2012;60(2):1331-1339. doi:http://dx.doi.org/10.1016/j.neuroimage.2012.01.088
10. Van Veen BD, van Drongelen W, Yuchtman M, Suzuki A. Localization of brain electrical activity via linearly constrained minimum variance spatial filtering. *IEEE transactions on bio-medical engineering*. 1997/09// 1997;44(9):867-80. doi:10.1109/10.623056
11. Muthuraman M, Raethjen J, Koirala N, et al. Cerebello-cortical network fingerprints differ among essential, Parkinson and mimicked tremors. *Brain*. 2018:In Press.
12. Makris N, Goldstein JM, Kennedy D, et al. Decreased volume of left and total anterior insular lobule in schizophrenia. *Schizophrenia Research*. 2006/04/01/ 2006;83(2):155-171. doi:https://doi.org/10.1016/j.schres.2005.11.020
13. Amjad AM, Halliday DM, Rosenberg JR, Conway BA. An extended difference of coherence test for comparing and combining several independent coherence estimates: theory and application to the study of motor units and physiological tremor. *Journal of neuroscience methods*. 1997/04/25/ 1997;73(1):69-79.
14. Rosenberg JR, Amjad AM, Breeze P, Brillinger DR, Halliday DM. The Fourier approach to the identification of functional coupling between neuronal spike trains. *Progress in Biophysics and Molecular Biology*. 1989 1989;53(1):1-31.
15. Muthuraman M, Hellriegel H, Hoogenboom N, et al. Beamformer source analysis and connectivity on concurrent EEG and MEG data during voluntary movements. *PloS one*. 2014;9(3):e91441. doi:10.1371/journal.pone.0091441
16. Haykin SS. *Kalman filtering and neural networks*. Wiley Online Library; 2001.
17. Wan EA, Nelson AT. Dual extended Kalman filter methods. *Kalman filtering and neural networks*. 2001:123-173.
18. Kaminski M, Ding M, Truccolo WA, Bressler SL. Evaluating causal relations in neural systems: granger causality, directed transfer function and statistical assessment of significance. *Biological cybernetics*. 2001/08// 2001;85(2):145-57.

19. Pagnotta MF, Plomp G. Time-varying MVAR algorithms for directed connectivity analysis: Critical comparison in simulations and benchmark EEG data. *PLoS ONE*. 2018;13(6):e0198846. doi:10.1371/journal.pone.0198846
20. Leistritz L, Pester B, Doering A, et al. Time-variant partial directed coherence for analysing connectivity: a methodological study. *Philos Trans A Math Phys Eng Sci*. Aug 28 2013;371(1997):20110616. doi:10.1098/rsta.2011.0616
21. Arnold N, Tapio S. Estimation of parameters and eigenmodes of multivariate autoregressive models. ACM; 2001. p. 27-57.
22. Tapio S, Arnold N. Algorithm 808: ARfit: a matlab package for the estimation of parameters and eigenmodes of multivariate autoregressive models. ACM; 2001. p. 58-65.
23. Haufe S, Nikulin VV, Muller KR, Nolte G. A critical assessment of connectivity measures for EEG data: a simulation study. *Neuroimage*. 2013/01/01/ 2013;64:120-33. doi:10.1016/j.neuroimage.2012.09.036
24. Thorne JD, De Vos M, Viola FC, Debener S. Cross-modal phase reset predicts auditory task performance in humans. *Journal of Neuroscience*. 2011;31(10):3853-3861.
25. Fiebelkorn IC, Foxe JJ, Butler JS, Mercier MR, Snyder AC, Molholm S. Ready, set, reset: stimulus-locked periodicity in behavioral performance demonstrates the consequences of cross-sensory phase reset. Research Support, N I H , Extramural
Research Support, Non-U S Gov't
Research Support, U S Gov't, Non-P H S. *J Neurosci*. 2011;31(27):9971-81.
26. Gonzalez-Escamilla G, Chirumamilla V, Meyer B, et al. Excitability regulation in the dorsomedial prefrontal cortex during sustained instructed fear responses: a TMS-EEG study. *Brain Stimulation*. March 6 2018;doi:http://dx.doi.org/10.1101/277806
27. Spaak E, de Lange FP, Jensen O. Local entrainment of alpha oscillations by visual stimuli causes cyclic modulation of perception. *Journal of Neuroscience*. 2014;34(10):3536-3544.
28. Gray MJ, Frey H-P, Wilson TJ, Foxe JJ. Oscillatory recruitment of bilateral visual cortex during spatial attention to competing rhythmic inputs. *Journal of Neuroscience*. 2015;35(14):5489-5503.
29. Stroh A, Adelsberger H, Groh A, et al. Making waves: initiation and propagation of corticothalamic Ca2+ waves in vivo. *Neuron*. 2013 2013;77(6):1136-50.
30. Buzsáki G, Moser EI. Memory, navigation and theta rhythm in the hippocampal-entorhinal system. *Nature neuroscience*. 2013 2013;16(2):130-138.
31. Pape H-C, Driesang RB. Ionic mechanisms of intrinsic oscillations in neurons of the basolateral amygdaloid complex. *J Neurophysiol*. 1998 1998;79(1):217-226.
32. Likhtik E, Gordon JA. Circuits in sync: decoding theta communication in fear and safety. *Neuropsychopharmacology: official publication of the American College of Neuropsychopharmacology*. 2014 2014;39(1):235-236.
33. Corbetta M, Shulman GL. Control of goal-directed and stimulus-driven attention in the brain. *Nature Reviews Neuroscience*. 2002 2002;3(3):201-215.
34. Fox MD, Corbetta M, Snyder AZ, Vincent JL, Raichle ME. Spontaneous neuronal activity distinguishes human dorsal and ventral attention systems. *Proceedings of the National Academy of Sciences*. 2006;103(26):10046-10051.
35. Papenberg G, Hämmerer D, Müller V, Lindenberger U, Li S-C. Lower theta inter-trial phase coherence during performance monitoring is related to higher reaction time variability: a lifespan study. *Neuroimage*. 2013 2013;83:912-920.
36. Cavanagh JF, Figueroa CM, Cohen MX, Frank MJ. Frontal theta reflects uncertainty and unexpectedness during exploration and exploitation. *Cereb Cortex*. 2012 2012;22(11):2575-86.

37. Ponjavic-Conte KD, Hambrook DA, Pavlovic S, Tata MS. Dynamics of distraction: competition among auditory streams modulates gain and disrupts inter-trial phase coherence in the human electroencephalogram. *PLoS One*. 2013;8(1):e53953.
38. Tiitinen H, Sinkkonen J, Reinikainen K, Alho K, Lavikainen J, Näätänen R. Selective attention enhances the auditory 40-Hz transient response in humans. *Nature*. 1993;364(6432):59-60.
39. Schaefer A, Kong R, Gordon EM, et al. Local-Global Parcellation of the Human Cerebral Cortex from Intrinsic Functional Connectivity MRI. *Cereb Cortex*. Sep 1 2018;28(9):3095-3114. doi:10.1093/cercor/bhx179
40. Yeo BT, Krienen FM, Sepulcre J, et al. The organization of the human cerebral cortex estimated by intrinsic functional connectivity. *J Neurophysiol*. Sep 2011;106(3):1125-65. doi:10.1152/jn.00338.2011
